# First draft genome sequence of a strain from the genus *Fusibacter* isolated from Salar de Ascotán in Northern Chile

**DOI:** 10.1186/s40793-017-0252-4

**Published:** 2017-07-24

**Authors:** Antonio E. Serrano, Lorena V. Escudero, Cinthya Tebes-Cayo, Mauricio Acosta, Olga Encalada, Sebastián Fernández-Moroso, Cecilia Demergasso

**Affiliations:** 1 Centro de Biotecnología, Universidad Católica del Norte, Antofagasta, Chile; 2 Centro de Investigación Científica y Tecnológica para la Minería, Antofagasta, Chile

**Keywords:** *Fusibacter*, Arsenic biogeochemistry, *Firmicutes*, *Clostridiales*, Hypersaline environment, Arsenate-reducing bacteria

## Abstract

*Fusibacter* sp*.* 3D3 (ATCC BAA-2418) is an arsenate-reducing halotolerant strain within the *Firmicutes* phylum, isolated from the Salar de Ascotán, a hypersaline salt flat in Northern Chile. This high-Andean closed basin is an athalassohaline environment located at the bottom of a tectonic basin surrounded by mountain range, including some active volcanoes. This landscape can be an advantageous system to explore the effect of salinity on microorganisms that mediate biogeochemical reactions. Since 2000, microbial reduction of arsenic has been evidenced in the system, and the phylogenetic analysis of the original community plus the culture enrichments has revealed the predominance of *Firmicutes* phylum. Here, we describe the first whole draft genome sequence of an arsenic-reducing strain belonging to the *Fusibacter* genus showing the highest 16S rRNA gene sequence similarity (98%) with *Fusibacter* sp. strain Vns02. The draft genome consists of 57 contigs with 5,111,250 bp and an average G + C content of 37.6%. Out of 4780 total genes predicted, 4700 genes code for proteins and 80 genes for RNAs. Insights from the genome sequence and some microbiological features of the strain 3D3 are available under Bioproject accession PRJDB4973 and Biosample SAMD00055724. The release of the genome sequence of this strain could contribute to the understanding of the arsenic biogeochemistry in extreme environments.

**Electronic supplementary material:**

The online version of this article (doi:10.1186/s40793-017-0252-4) contains supplementary material, which is available to authorized users.

## Introduction

Salt flats or *salares* are one of the most interesting biomes on earth [1]. Based on their hypersaline conditions, prokaryotes have evolved to develop biochemical processes with potential applications in biotechnology [2], providing also opportunities for biosignature detection on other planets [3]. Due to geological, climatic and geomorphological factors, dozens of endorheic basins are located in Northern Chile including evaporitic bodies and saline lakes. Brines and crusts of these saline deposits are enriched in arsenic [4].

The Salar de Ascotán [5] is an athalassohaline environment located at the bottom of a tectonic basin surrounded by volcanic systems in east-west direction, including some active volcanoes with altitudes from 5000 to 6000 m.a.s.l. [6]. The saline crusts are mainly composed of chlorides (halite) and sulfates (gypsum) to economic boron-bearing minerals associated with significant amounts of arsenic sulfides [5], with the arsenic concentrations the highest found in the area [7].

In order to understand the bacterial role in the arsenic biogeochemical cycle at circumneutral pH, several sampling expeditions to Salar de Ascotán, in the Chilean High-Andes, have been taken since 2000. The microbial diversity of this salt flat was first analyzed [7], then enrichment [8], isolation and sequencing efforts [9] as well as geochemical in situ investigations wer performed [10]. In addition, the distribution of genes for the As (III) oxidation (*aioA*), As (V) detoxifying respiration (*arrA*), As detoxification (*arsC*), and As (III) extrusion (*acr3*) was explored in Salar de Ascotán and other natural environments in Northern Chile with arsenic concentrations spanning six orders of magnitude. The abundance of *Firmicutes*-like *arsC* genes compared to the *Enterobacterial*-like *arsC* genes in these environments suggested an important role of thioredoxin and the *Firmicutes* phylum in the local As biogeochemistry [11].

*Fusibacter* is a minor genus into the *Clostridiales* order within the *Firmicutes* phylum. Currently, it comprises four Gram-positive species with validly published names. This group started with the discovery of the thiosulfate-reducing bacterium *Fusibacter paucivorans* *,* being the most studied, isolated from oil-producing wells [12]. *Fusibacter tunisiensis* was isolated from an anaerobic reactor used to treat olive-mill wastewater [13]. Recently, *Fusibacter bizertensis* was identified from a corroded kerosene storage tank [14], and more recently, *Fusibacter fontis* was the first species of this genus isolated from a natural environment [15]. In general terms, the reported members of this genus are fermentative and halotolerant anaerobes. Moreover, these species share sulfur-reducing features capable of generating sulfide starting from elemental sulfur [13, 15] or thiosulfate [12, 13] sources. To date, a whole-genome sequence has not been reported for any species within this genus.

Here, we report the first draft genome of a strain of *Fusibacter* plus some microbiological properties of this halotolerant isolate, recovered from a saline environment in Northern Chile. The strain was deposited as *Fusibacter* *sp.* 3D3 as ATCC BAA-2418 because we are still running the necessary tests and deposits to describe the isolate as a new species and “*Fusibacter* *ascotence”* is the proposed species name.

This report contributes to a better understanding of the ecophysiology of extreme halotolerant microorganisms inhabiting saline environments and their role in the arsenic biogeochemistry.

## Organism information

### Classification and features

**Fig. 1:**
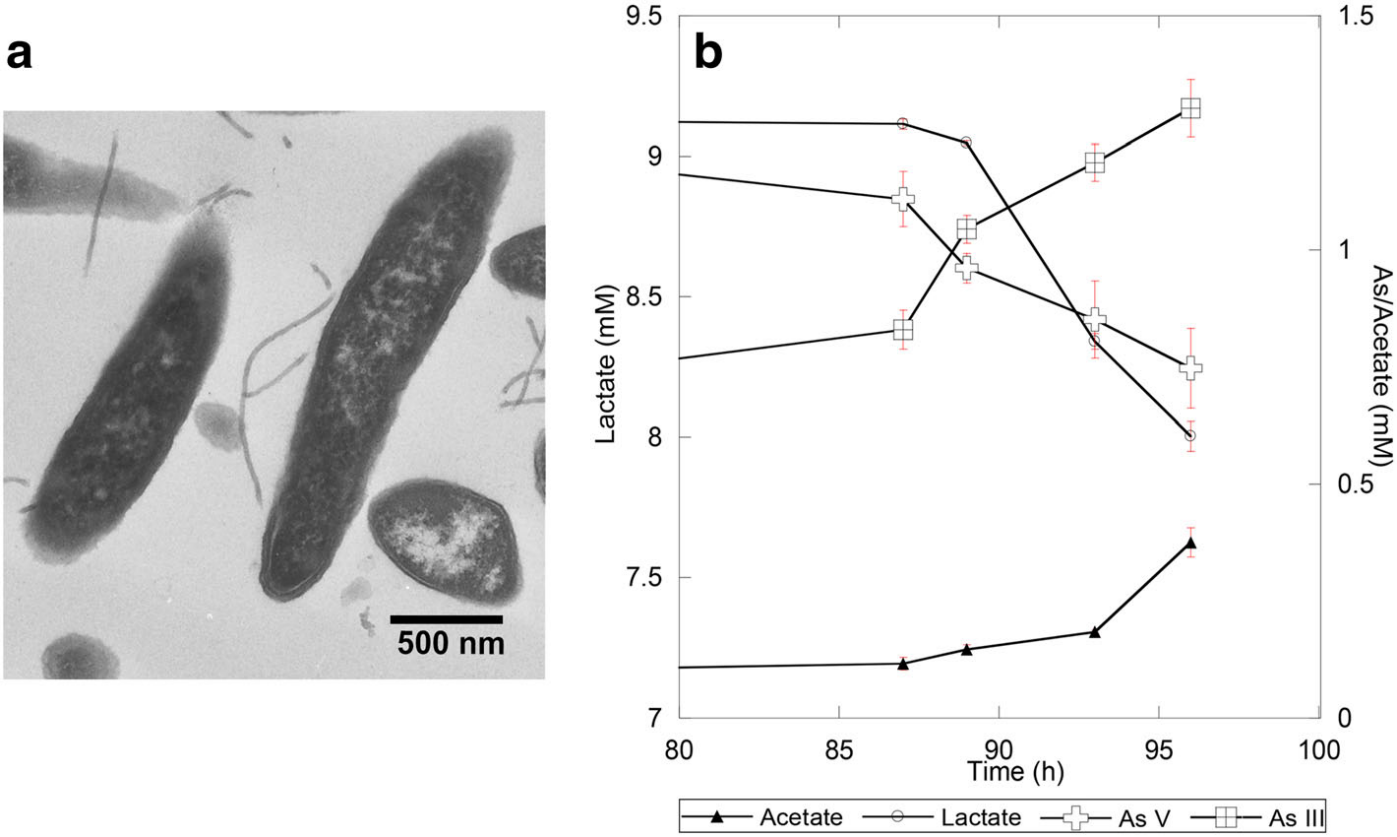
Isolation of *Fusibacter* sp. 3D3. **a** Transmission electron micrograph of bacterial cells filled with electron dense granules of variable density (Bar = 500 nm). **b** Arsenic speciation, lactate and acetate measurements of *Fusibacter* sp. strain 3D3 culture in Newman’s modified medium with 20 mM lactate, 10 mM sulphate, 2 mM arsenate, 0.1% (*w*/*v*) yeast extract, and 1 mM cysteine versus time. All error bars represent the standard error of the mean of triplicate cultures

*Fusibacter* sp. 3D3 is an indigenous strain of the Salar de Ascotán hypersaline sediments isolated at the Centro de Biotecnología, Universidad Católica del Norte, Antofagasta, Chile. Enrichment, isolation, and growth experiments were performed in a fresh Newman-modified minimal medium [7] containing, 1% (*w*/*v*) NaCl, 0.1% (*w*/*v*) yeast extract, and 1 mM cysteine adjusted to pH 7.0. After autoclaving, 10 mM lactate as electron donor, and 20 mM sodium sulfate and 2 mM sodium arsenate as electron acceptors were added in order to complete 20 mL of medium into 50 mL-anaerobic-bottles (Supelco). The strain was incubated in an anaerobic chamber (Airlock, Coydrive), in dark, at 30 °C, under N_2_:CO_2_:H_2_ gas atmosphere (80:15:5, *v*/v) up to 10 days. The pure colonies were obtained by inclined tubes of agar prepared with the mentioned Newman modified medium plus 2% (*w*/*v*) agar incubated under anaerobic conditions at 30 °C. Single yellow colonies were restreaked several times to obtain pure isolates and then were transferred to the liquid medium. Transmission electron microscopy revealed rod-shaped cells (0.4 μm × 3-10 μm) (Fig. 1a).

Arsenate reduction was tested by inoculation of 1 × 10^−6^ cells mL^−1^ into 20 mL of fresh Newman-modified medium under incubation conditions described above. An abiotic control was carried out in sterile medium without inoculum. Growth curves were performed in triplicate and monitored by counting chambers (0.01 mm × 0.0025 mm^2^, Neubauer, Marienfeld). Samples were acquired periodically, then centrifuged (15,000×g; 10 min) to remove cells, and finally filtered through a 0.2 μm cellulose filter. The filtered supernatant was sealed and refrigerated at 4 °C to preserve arsenic speciation until analysis. As (V) and As (III) concentrations were measured with a mobile phase of 10 mM acid phosphate at 6.25 pH by Millennium Excalibur HPLC System (PS Analytical, Orpington, UK). To quantify lactate and acetate, each filtrate was injected in a Dionex IonPac AS11-HC column to run a high-performance liquid chromatography (Thermo Scientific model 3200) with an isocratic concentration of KOH. Arsenate reduction and simultaneous lactate consumption were evidenced (Fig. 1b). Arsenate reduction has not been reported in the other members of the *Fusibacter* genus.

**Fig. 2:**
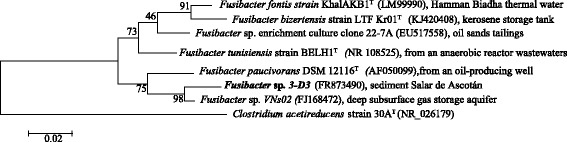
Phylogenetic tree based on the 16S rRNA gene sequences highlighting the position of *Fusibacter* sp. strain 3D3 relative to other type and non-type strains of the genus *Fusibacter*. The Genbank database accession codes (in *brackets*) is indicated. Bootstrap values for 500 replicates are indicated at the nodes. Scale bar 0.02 substitutions per nucleotide position

The Initial identification of strain 3D3 was performed by 16S rRNA gene amplification using a previously described method [7]. The 16S rRNA sequences of strain 3D3 clustered with type strains of *Fusibacter* species when those were aligned using Clustal W and manually corrected. A phylogenetic tree was constructed using neighbor-joining, maximum-parsimony, and maximum likelihood algorithms with bootstrap values of 500 replicates using the MEGA program version 6.22. Phylogenetic analysis of the 16S rRNA sequence indicated that the strain 3D3 belongs to the genus *Fusibacter* and exhibits a similarity of 98% with *Fusibacter* sp. Vns02, and 95% with both *Fusibacter paucivorans* and *Fusibacter tunisiensis* (Fig. 2).

RapID™ NF Plus and RapID™ One (Thermo Scientific), two qualitative micromethods employing conventional and chromogenic substrates for the biochemical features identification were performed following the manufacturer’s instructions (Table 1). A single colony was inoculated into the given fluid and incubated for 24 h at 30 °C. Then, the inoculation fluid was transferred to the corresponding panel. The interpreted results were entered into the electronic RapID code database (ERIC electronic compendium, version 1.0.771, patch 0613). Comparing with the ERIC™ database, our results failed to identify our isolate (Table 1).

**Table 1:** Biochemical analyses of Fusibacter sp. strain 3D3

Analysis	Test Code	Reactive ingredient	Result
Amino acids hydrolysis	ADH	Arginine	+
	ODC	Ornitine	+
	LDC	Lysine	-
Enzymatic hydrolysis of arylamide	PRO	Proline-β-naphthylamide	+
	PYR	Pyrrolidine-β-naphthylamide	+
	GGT	γ-Glutamyl-β naphthylamide	-
	TRY	Tryptophan-β- naphthylamide	-
	BANA	N-Bencyl-arg-β-naphthylamide	-
Enzymatic hydrolysis of glucoside	PHS	N-nitrophenyl-phosphoester	+
	NAG	N-nitrohenyl-N-acetyl-β-D-glucosaminide	+
	αGLU	N-nitrophenyl-α-D-glucoside	-
	βGLU	N-nitrophenyl-β-D-glucoside	-
	ONPG	N-nitrophenyl-β-D-galactoside	-
	GUR	N-nitrophenyl-β-D-glucuronide	-
	βXYL	N-nitrophenyl-β-D-xyloside	-
Carbohydrate utilization	KSF	Sugar aldehyde	-
	SBL	Sorbitol	-
	ADON	Adonitol	-
	EST	Thiol assay	+
	IND	Tryptophan assay	-
	MAL	Malonate assay	+
	GLU	Glucose assay	O
	NO_3_	Nitrate assay	+
	URE	Hydrolysis of urea	-
	OXI	Cytochrome oxidase	-

*O Oxidation*

Analyses were performed utilizing RapID^TM^ NF Plus and RapID^TM^ One kits (Thermo Scientific)

## Genome sequencing information

### Genome project history

Based on its phylogenetic position and 16S rRNA similarity, *Fusibacter* *sp*. 3D3 (Taxonomy ID: 1,048,380) was previously submitted to NCBI in 2010 (Gene Bank 16S rRNA gene: FR873490.1) (Fig 2). Later, in 2013, it was deposited as *Fusibacter* sp. 3D3 in ATCC BAA-2418, being the first strain of this genus coming from an extreme arsenic bearing and saline biotope (Table 2). This organism was selected for genome sequencing based on its interesting phenotypic characteristics. Recently, in 2016, the submission of the whole shotgun project assembled as a draft genome was performed to the DNA Data Bank of Japan under the Bioproject accession number PRJDB4973 and Biosample number SAMD00055724 (ID 573014). This Whole Genome Shotgun project has been deposited at GenBank under the accession BDHH00000000. The version described in this paper is the first version, BDHH01000000 [16]. Table 3 presents the project information and its association with MIGS version 2.0 compliance [17].

**Table 2:** Classification and general features of *Fusibacter* sp. strain 3D3 [18]

MIGS ID	Property	Term	Evidence code^a^
	Classification	Domain *Bacteria*	TAS [31]
		Phylum *Firmicutes*	TAS [32]
		Class *Clostridia*	TAS [33]
		Order *Clostridiales*	TAS [34]
		Family *Clostridiales Family XII*	TAS [33]
		Genus *Fusibacter*	TAS [12]
		Specie *Fusibacter sp. 3D3*	IDA
		Strain: *3D3 (Accession # FR873490.1)*	
	Gram stain	Positive	IDA
	Cell shape	Point end rod	IDA
	Motility	Motile	IDA
	Sporulation	Spore forming	NAS
	Temperature range	20 -35 °C	IDA
	Optimum temperature	30 °C	IDA
	pH range; optimum	5–9; 7	IDA
	Carbon source	Lactate, Tryptone, Glucose	IDA
MIGS-6	Habitat	Salt-flat sediment	IDA
MIGS-6.3	Salinity	1% (*w*/*v*) NaCl	IDA
MIGS-22	Oxygen requirement	Anaerobe	IDA
MIGS-15	Biotic relationship	Free-living	IDA
MIGS-14	Pathogenicity	Non-pathogen	NAS
MIGS-4	Geographic location	Ascotán salt flat, Antofagasta region, Chile	IDA
MIGS-5	Sample collection	21-Sep-2010	IDA
MIGS-4.1	Latitude	21°36′06.2″ S	IDA
MIGS-4.2	Longitude	68°18′28.3″ W	IDA
MIGS-4.4	Altitude	3748 m.a.s.l.	IDA

^a^Evidence codes - *IDA* inferred from direct assay, *TAS* traceable author statement (i.e., a direct report exists in the literature), *NAS* non-traceable author statement (i.e., not directly observed for the living, isolated sample, but based on a generally accepted property for the species, or anecdotal evidence). These evidence codes are from the Gene Ontology project [35]

**Table 3:** Project information

MIGS ID	Property	Term
MIGS 31	Finishing quality	Draft
MIGS-28	Libraries used	Nextera Illumina
MIGS 29	Sequencing platforms	MiSeq Illumina
MIGS 31.2	Fold coverage	50×
MIGS 30	Assemblers	Newbler v2.0.01.14.
MIGS 32	Gene calling method	Glimmer
	Locus Tag	F3D3
	Genbank ID	BDHH00000000
	GenBank Date of Release	2016-09-05
	GOLD ID	GP0193989
	BIOPROJECT	PRJDB4973
MIGS 13	Source Material Identifier	3D3
	Project relevance	Arsenic biogeochemical cycle, Territorial biodiversity, Bionanotechnology, Bioremediation, Biogeochemistry

### Growth conditions and genomic DNA preparation

*Fusibacter* *sp*. strain 3D3 was grown anaerobically on fresh Newman [18] modified medium and conditions of incubation described above. DNA was extracted using High Pure Template Preparation Kit (Roche, Germany), according to the manufacturer instructions. Both quantity and quality of the genomic DNA were measured using a NanoDrop ND-1000 spectrophotometer (Thermo-Fisher Scientific Inc.) and analyzed by DGGE (200 C. B. S. Scientific Company), respectively. The purity of strain 3D3 was confirmed by a single band in the DGGE profile.

### Genome sequencing and assembly

The genome of *Fusibacter* sp. 3D3 was sequenced on an Illumina MiSeq platform at Molecular Research Laboratory (MR. DNA, Shallowater, TX). The library for each sample was prepared using a Nextera DNA Sample Preparation Kit (Illumina), following the manufacturer’s instructions. Sequencing of 2 × 300-bp paired-end reads allowed for an estimate of 20,000 output with an average coverage over 50 times fold. The assemblage of quality-filtered reads was executed by MR-DNA for the complete genome sequence. As a result, the draft genome of ~5.1 Mbp size was generated. Reads were assembled de novo using Newbler v2.0.01.14. The final draft assembly contained 57 contigs identifying 4780 genes using RAST [19].

### Genome annotation

Genes were predicted using Glimmer 3.02 [20] as part of the RAST annotation pipeline using SEED platform for 4780 features identified. Whole RNA genes were also predicted by the same annotation platform [21]. The predicted protein coding genes were analyzed for the presence of signal peptides using SignalP 4.1 Server [22]. TMHMM Server v. 2.0 was utilized for prediction of transmembrane helices in proteins [23]. Geneious 7.1.9 (Biomatters) software was used to analyze COGs utilizing the BLAST COG database (Table 4). Pfam domains were computed using InterProScan 5.19-58.0 [24]. CRISPRs repeats were found submitting the contigs to the CRISPRs Finder web server [25].

**Table 4:** Number of genes associated with general COG functional categories

Code	Value	% age	Description
J	211	4.5	Translation, ribosomal structure and biogenesis
A	0	0.0	RNA processing and modification
K	394	8.4	Transcription
L	321	6.8	Replication, recombination and repair
B	2	0.0	Chromatin structure and dynamics
D	53	1.1	Cell cycle control, Cell division, chromosome partitioning
V	125	2.7	Defense mechanisms
T	280	6.0	Signal transduction mechanisms
M	175	3.7	Cell wall/membrane biogenesis
N	136	2.9	Cell motility
U	36	0.8	Intracellular trafficking and secretion
O	129	2.7	Posttranslational modification, protein turnover, chaperones
C	262	5.6	Energy production and conversion
G	286	6.1	Carbohydrate transport and metabolism
E	421	9.0	Amino acid transport and metabolism
F	85	1.8	Nucleotide transport and metabolism
H	136	2.9	Coenzyme transport and metabolism
I	102	2.2	Lipid transport and metabolism
P	186	4.0	Inorganic ion transport and metabolism
Q	68	1.4	Secondary metabolites biosynthesis, transport and catabolism
R	462	9.8	General function prediction only
S	331	7.0	Function unknown
-	499	10.6	Not in COGs

The total is based on the total number of protein coding genes in the genome

## Genome properties

The draft genome for *Fusibacter* sp. 3D3 contained 5,111,250 nucleotides with an average G + C content of 37.6% (Table 5). From 4780 genes, 4700 were predicted protein coding, and 80 RNA coding genes (12 rRNA, and 62 tRNA genes). The putative function was assigned to 63.1% of the genes, while the remaining genes were annotated as hypothetical proteins. The distribution of genes in COGs functional categories is presented in Table 4.

**Table 5:** Genome statistics

Attribute	Value	% of Total
Genome size (bp)^a^	5,111,250	100.0
DNA coding (bp)	4,450,431	87.1
DNA G + C (bp)	1,921,825	37.6
DNA scaffolds	57	100.0
Total genes	4780	100,0
Protein coding genes	4700	98,3
RNA genes^b^	80	1.7
Pseudo genes	n.d	n.d
Genes in internal clusters^c^	n.d	n.d
Genes with function prediction	3156	67.1
Genes assigned to COGs	4201	89.3
Genes with Pfam domains	3711	77.4
Genes with signal peptides	254	5.3
Genes with transmembrane helices	1219	25.8
CRISPR repeats^d^	10	0.2

^a^The total is based on either the size of the genome in base pairs or the total number of genes in the annotated genome

^b^Includes tRNA, mRNA, rRNA

^c^ *n.d.* Not determined

^d^Including confirmed and questionable

## Insights from the genome sequence

Similarity analysis of genes involved in the arsenic metabolism indicated that the closest available genome of strain 3D3 in the database was *Clostridium sticklandii* [26], which belongs to the *Clostridiales* family as well. The subsystem information approach to genome annotation performed by RAST/SEED [27] confirmed the relation to other members in the *Clostridiales* order (Table 6). Arsenic detoxification genes are clearly present in *Fusibacter* sp. 3D3 genome, however, genes coding for arsenate respiratory reductases (*arr*) and arsenite oxidases (*aio*) have a very low percentage of similarity with genes coding for the enzymes evidenced at protein level. The *arsC* gene sequence identified in the *Fusibacter* sp. genome was clustered inside the *Firmicutes*-like *arsC* gene clade whose predominance has been reported in Salar de Ascotán [11].

**Table 6:** BLAST results of predicted and best-scored proteins related to arsenic in the Fusibacter sp. strain 3D3 genome

Subsystem	Gene	Functional role	Contig/ CDS	Closest Protein Homology^a^			
				Specie	%	E-value	UniProt
Anaerobic reductases	*aprB*	Adenylylsulfate reductase beta-subunit	2/3276	*Roseburia* sp. CAG:100	65	3 × 10^-22^	R7R6L1
Arsenic related genes	*arsA*	Arsenical pump-driving ATPase (EC 3.6.3.16)	39/1529	*Clostridium* sp*. s*train BNL1100	83	1 × 10^-59^	H2J8R6
	*arsC*	Arsenate reductase (EC 1.20.4.1)	49/1898	*Amphibacillus xylanus*	71	2 × 10^-51^	K0J2A1
	*arsR*	Arsenical resistance operon repressor	49/1984	*Desulfitobacterium hafniense*	68	3 × 10^-49^	Q24NC4
	*arrA*	Respiratory arsenate reductase, Mo binding subunit	31/1301	*Shewanella* sp. strain ANA-3	29	1.4	Q7WTU0
	*arrB*	Respiratory arsenate reductase, FeS subunit	52/2102	*Shewanella* sp. strain ANA-3	42	2 × 10^-5^	Q7WTT9
	*aCR3*	Arsenical-resistance protein	49/1897	*Clostridium sticklandii*	86	0	E3PWS9
	*arsD*	Arsenical resistance operon trans-acting repressor	14/298	*Clostridium botulinum*	27	0,39	A5HZU7
	*arsR*	Arsenical resistance operon repressor	39/1526	*Dehalobacter* sp. strain DCA	52	2 × 10 ^-43^	K4LCR7
	*arsR2*	Transcriptional regulator, ArsR family	72/3456	*Methylobacterium extorquens*	29	1 × 10^-4^	C5B3N6
	*aoxS*	Periplasmic sensor signal transduction his-kinase	79/3664	*Alkaliphilus oremlandii* strain OhILAs	46	0	A8MKM5
	*aoxR*	Transcriptional regulator	79/3663	*Alkaliphilus oremlandii*	58	5 × 10^-178^	A8MKM4
	*arsM*	S-adenosylmethionine-dependent methyltransferas	65/3260	*Paenibacillus polymyxa* strain M1	61	5 × 10^-85^	E3E8M9
	*arxB*	4Fe-4S binding domain-containing protein	17/387	*Ectothiorhodospira* sp. strain PHS-1	29	0.002	H1G3R8
	*arxA*	Anaerobic arsenite oxidase	17/353	*Ectothiorhodospira* sp. strain PHS-1	33	0.92	H1G3R7
	*arxC*	Polysulfide reductase, NrfD	49/1666	*Sulfuricella denitrificans* strain skB26	31	1.6	S6AE44
Electron Transport	*cymA*	Cytochrome c-type protein	24/1193	*Shewanella putrefaciens*	31	0.25	P95832
	*rnfA*	Electron transport complex protein RnfA	52/2101	*Eubacterium acidaminophilum*	77	5 × 10^-95^	W8TJP4
	*rnfB*	Electron transport complex protein RnfB	52/2102	*Alkaliphilus metalliredigens*	63	4 × 10^-160^	A6TQH4
	*rnfC*	Electron transport complex protein RnfC	52/2097	*Clostridium sticklandii*	64	0	E3PRL8
	*rnfD*	Electron transport complex protein RnfD	52/2098	*Eubacterium acidaminophilum*	64	5 × 10^-135^	W8T3U4
	*rnfE*	Electron transport complex protein RnfE	52/2100	*Clostridium bartlettii* CAG:1329	70	5 × 10^-92^	R5Y4N2
	*rnfG*	Electron transport complex protein RnfG	52/2099	*Clostridium sordellii* VPI 9048	43	1 × 10^-44^	T0CLK2
Oxidoreductase	*trx*	Thioredoxin reductase/ FAD/NAD-binding	6/2715	*Youngiibacter fragilis*	76	0	V7I8R3
	*ahpC*	Thioredoxin	64/3082	*Clostridium sticklandii* strain ATCC 12662	87	8 × 10^-109^	E3PTE6

^a^Percentage (%) of identity by alignment overview UNIPROTKB is indicated

Subsystem information was obtained by RAST/SEED viewer v2.0

**Fig. 3:**
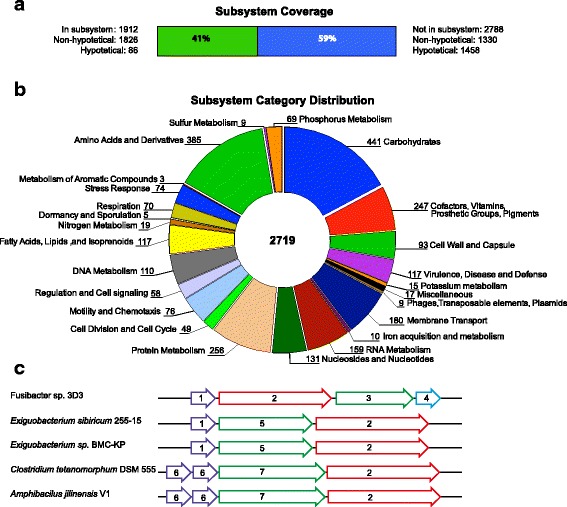
Summary of subsystems identified by RAST/SEED. **a** Subsystem coverage. 41% of the whole CDS were identified in subsystems. **b** Subsystem category distribution of all non-hypothetical CDS. No photosynthesis and secondary metabolites CDS were assigned. **c** Assigned CDS for arsenic metabolism in the *Fusibacter* sp. strain 3D3 genome comparing to closely related species. Diagram of the chromosomal region (Contig 49) of the arsenic-related genes compared with five microorganisms. 1. Arsenical resistance operon repressor ArsR, 2. Pyridine nucleotide-disulfide oxidoreductase NADH dehydrogenase (EC 1.6.99.3), 3. Arsenical resistance protein ACR3, 4. Arsenate reductase ArsC (EC 1.20.4.1), 5. arsenical efflux pump membrane protein ArsB, 6. Arsenical resistance operon trans-acting repressor ArsD, 7. Arsenical pump-driving ATPase ArsA (EC 3.6.3.16)

In the vicinity of the ArsC coding gene is the gen F3D3_RS05420. This piqued our interest and, in a deeper analysis, we found that F3D3_RS05420 codify for pyridine nucleotide-disulfide oxidoreductase NADH dehydrogenase (accession number: WP_069871897). The preliminary information indicates that the protein encoded by the gen F3D3_RS05420 is part of a new family of proteins of unknown function. However, the genomic context shows us some clues to formulate a hypothesis. By means of comparative genomics we identify two common components accompanying genes like F3D3_RS05420: A) genes codifying for transcription regulators and, B) genes codifying for arsenical transporters (Fig. 3c). This could be an indicative of a possible role in the response to stress by As. The multiple sequence alignment carried out using MUSCLE application [28] in CLC Genome Workbench 8.0 (Qiagen) shows that the protein is distributed in the *Firmicutes* bacteria and it is strongly conserved (Additional file 1: Figure S1).

The protein architecture of WP_069871897 shows a CoA-disulfide reductase domain (TIGR03385) and a rhodanese domain (PFAM00581). A rhodanese domain is also present in the ACR2 protein of *Saccharomyces cerevisiae* which also has arsenate reductase activity [29]. The catalytic loop of the rhodanese domain has two known configurations, a short version with four residues to accommodate sulfur or selenium atoms and an extended version with five residues to accommodate an arsenic or phosphorus atoms [30]. The architecture of WP_069871897 and related proteins suggest a role similar to ACR2, namely arsenic reductase. However, the comparison between the catalytic loops of ACR2 (Q06597) “CTGSKNRG” with the “CNKGVTGN” of WP_069871897 does not show an apparent similitude, which makes it difficult to extrapolate the activity of ACR2 with WP_069871897. In addition, the presence of the *arsC* gene in the compared genomes (Fig. 3c), but not in the same genomic context, suggests that the WP_069871897 and related proteins does not substitute the ArsC activity. Then, it remains a challenge to the scientific community to answer if the proteins similar to WP_069871897 are a new kind of arsenic reductase or if they are in some way involved with the response to arsenic stress.

## Conclusions

The 5.11 Mbp draft genome sequence of *Fusibacter* sp. 3D3 is arranged in 57 contigs, being the first *Fusibacter* draft genome published. It potentially includes 4700 protein-coding genes, 67.1% of which were assigned to function prediction. 80 RNA genes partitioned in 12 rRNA and 62 tRNAs were identified. The release of the genome sequence of this strain will provide new insights into arsenic reduction processes in hypersaline biomes and further understanding of the mechanisms used by halophile bacteria to endure high osmotic stress in natural and industrial saline environments.

## Additional file

Additional file 1: Figure S1. CLUSTAL multiple sequence alignment of proteins related to WP_069871897 of *Fusibacter* sp. strain 3D3. (ALN 76400 bytes). (DOCX 38 kb)

## Abbreviations

m.a.s.l: Meters above sea level
MIGS: The minimum information about a genome sequence
RAST: Rapid annotations using subsystems technology

## References

[1] Demergasso C, Dorador C, Meneses D, Blamey J, Cabrol N, Escudero L (2010). Prokaryotic diversity pattern in high-altitude ecosystems of the Chilean Altiplano. J Geophys Res Biogeosci.

[2] Bull AT (2013). Asenjo J a. Microbiology of hyper-arid environments: recent insights from the Atacama Desert, Chile. Antonie Van Leeuwenhoek.

[3] Parro V, de Diego-Castilla G, Moreno-Paz M, Blanco Y, Cruz-Gil P, Rodríguez-Manfredi JA (2011). A microbial oasis in the hypersaline Atacama subsurface discovered by a life detector chip: implications for the search for life on Mars. Astrobiology.

[4] Risacher F, Fritz B (2009). Origin of salts and brine evolution of Bolivian and Chilean salars. Aquat Geochem.

[5] Chong G, Pueyo JJ, Demergasso C (2000). The borate deposits in Chile. Rev Geol Chile.

[6] Chong G (1984). Die Salare in Nordchile-Geologie. Struktur und geochimie Goetekt Forsch.

[7] Demergasso CS, Guillermo CD, Lorena EG, Mur JJP, Pedrós-Alió C (2007). Microbial precipitation of arsenic sulfides in Andean salt flats. Geomicrobiol J.

[8] Lara J, Escudero González L, Ferrero M, Chong Díaz G, Pedrós-Alió C, Demergasso C (2012). Enrichment of arsenic transforming and resistant heterotrophic bacteria from sediments of two salt lakes in Northern Chile. Extremophiles.

[9] Valdés N, Rivera-Araya J, Bijman J, Escudero L, Demergasso C, Fernández S (2014). Resistant Gammaproteobacterium isolated from a salt flat. Genome Announc.

[10] Nuñez C. Estudio de los procesos Biogeoquímicos que controlan la distribución del arsénico en el Salar de Ascotán y Gorbea. Antofagasta: Master thesis, Universidad Catolica del Norte; 2011.

[11] Escudero LV, Casamayor EO, Chong G, Pedrós-Alió C, Demergasso C (2013). Distribution of microbial arsenic reduction, oxidation and extrusion genes along a wide range of environmental arsenic concentrations. PLoS One.

[12] Ravot G, Magot M, Fardeau ML, Pateli BKC, Garcia JL, Ollivier B (1999). *Fusibacter paucivorans* gen. nov., sp. nov., an anaerobic, thiosulfate-reducing bacterium from an oil-producing well. Int J Syst Bacteriol.

[13] Ben Hania W, Fraj B, Postec A, Fadhlaoui K, Hamdi M, Ollivier B (2012). *Fusibacter tunisiensis* sp. nov., isolated from an anaerobic reactor used to treat olive-mill wastewater. Int J Syst Evol Microbiol.

[14] Smii L, Ben Hania W, Cayol J-L, Joseph M, Hamdi M, Ollivier B (2015). *Fusibacter bizertensis* sp. nov., isolated from a corroded kerosene storage tank. Int J Syst Evol Microbiol.

[15] Fadhlaoui K, Ben Hania W, Postec A, Fauque G, Hamdi M, Ollivier B (2015). *Fusibacter fontis* sp. nov., a sulfur-reducing, anaerobic bacterium isolated from a mesothermic Tunisian spring. Int J Syst Evol Microbiol.

[16] Mashima J, Kodama Y, Kosuge T, Fujisawa T, Katayama T, Nagasaki H (2016). DNA data bank of Japan (DDBJ) progress report. Nucleic Acids Res.

[17] Field D, Garrity G, Gray T, Morrison N, Selengut J, Sterk P (2008). The minimum information about a genome sequence (MIGS) specification. Nat Biotechnol.

[18] Newman DK, Beveridge TJ, Morel F (1997). Precipitation of arsenic Trisulfide by *Desulfotomaculum auripigmentum*. Appl Environ Microbiol.

[19] Aziz RK, Bartels D, Best AA, DeJongh M, Disz T, Edwards RA (2008). The RAST server: rapid annotations using subsystems technology. BMC Genomics.

[20] Delcher AL, Bratke KA, Powers EC, Salzberg SL (2007). Identifying bacterial genes and endosymbiont DNA with glimmer. Bioinformatics.

[21] Overbeek R, Olson R, Pusch GD, Olsen GJ, Davis JJ, Disz T (2014). The SEED and the rapid annotation of microbial genomes using subsystems technology (RAST). Nucleic Acids Res.

[22] Petersen TN, Brunak S, von Heijne G, Nielsen H (2011). SignalP 4.0: discriminating signal peptides from transmembrane regions. Nat Methods.

[23] Krogh A, Larsson B, von Heijne G, Sonnhammer ELL (2001). Predicting transmembrane protein topology with a hidden Markov model: application to complete genomes. J Mol Biol.

[24] Jones P, Binns D, Chang HY, Fraser M, Li W, McAnulla C (2014). InterProScan 5: genome-scale protein function classification. Bioinformatics.

[25] Grissa I, Vergnaud G, Pourcel C, Bland C, Ramsey TL, Sabree F (2007). CRISPRFinder: a web tool to identify clustered regularly interspaced short palindromic repeats. Nucleic Acids Res.

[26] Fonknechten N, Chaussonnerie S, Tricot S, Lajus A, Andreesen JR, Perchat N (2010). *Clostridium sticklandii*, a specialist in amino acid degradation:revisiting its metabolism through its genome sequence. BMC Genomics.

[27] Overbeek R, Begley T, Butler RM, Choudhuri JV, Chuang HY, Cohoon M (2005). The subsystems approach to genome annotation and its use in the project to annotate 1000 genomes. Nucleic Acids Res.

[28] Edgar RC (2004). MUSCLE: a multiple sequence alignment method with reduced time and space complexity. BMC Bioinformatics.

[29] Mukhopadhyay R, Rosen BP (1998). Saccharomyces Cerevisiae ACR2 gene encodes an arsenate reductase. FEMS Microbiol Lett.

[30] Bordo D, Bork P (2002). The rhodanese/Cdc25 phosphatase superfamily. Sequence-structure-function relations. EMBO Rep.

[31] Woese CR, Kandler O, Wheelis ML (1990). Towards a natural system of organisms: proposal for the domains archaea, bacteria, and eucarya. Proc Natl Acad Sci U S A.

[32] Ludwig W, Schleifer KH, Whitman WB, De Vos P, Garrity G, Jones D, Krieg NR, Ludwig W, Rainey FA, Schleifer KH, Whitman WB (2009). Revised road map to the phylum Firmicutes. Bergey's manual of systematic bacteriology.

[33] Yutin N, Galperin MY (2013). A genomic update on clostridial phylogeny: gram-negative spore formers and other misplaced clostridia. Environ Microbiol.

[34] Prévot AR.. Dictionnaire des Bactéries Pathogenes In: Hauduroy P, Ehringer G, Guillot G, Magrou J, Prevot AR, Rosset A, Urbain A, editors. 2nd ed. Masson: Paris; 1953.

[35] Ashburner M, Ball CA, Blake JA, Botstein D, Butler H, Cherry JM (2000). Gene ontology: tool for the unification of biology. Nat Genet.

