# Supplementary material for: First draft genome sequence of a strain from the genus Fusibacter isolated from Salar de Ascotán in Northern Chile
Source: Stand Genomic Sci. 2017 Jul 24;12:43. doi: 10.1186/s40793-017-0252-4 (PMC5525254; doi:10.1186/s40793-017-0252-4)
Supplement: Additional file 1: Figure S1. — CLUSTAL multiple sequence alignment of proteins related to WP_069871897 of Fusibacter sp. strain 3D3. (ALN 76400 bytes). (DOCX 38 kb) [file 40793_2017_252_MOESM1_ESM.docx]

Supplementary Material

First draft genome sequence of a strain from the genus [*Fusibacter*](http://doi.org/10.1601/nm.4203) isolated from **Salar de Ascotán** in northern [Chile](https://www.google.com/maps/place/Chile)

**Figure S1. CLUSTAL multiple sequence alignment of proteins related to WP_069871897 of Fusibacter sp. strain 3D3. (ALN 76400 bytes)**

CLUSTAL multiple sequence alignment

OHD09048.1 --MKIVIIGAVAAGTSVAAKARRNSEEAQIVVYEKDTDISYSVCGLPYYIAGSEIKREKL

WP_038668288.1 --MRIIIIGSVAAGTSVAAKARRNNIDNEIVIYEKDRDISYSVCGLPYYIGEEYISRENL

WP_026825315.1 --MKLVIIGSVAAGTSVAAKARRNNEDAQITIYDRDYDISYSGCGIPYYVGGEVESRDDL

WP_074036424.1 --MKLVIIGSVAAGTSVAAKARRNNEDAQITIYDRDYDISYSGCGIPYYVGGEVESRDDL

WP_047794995.1 --MKLVIIGSVAAGTSVAAKARRNNEDAQITIYDRDYDISYSGCGIPYYVGGEVESRDDL

WP_074036076.1 --MKLVIIGSVAAGTSVAAKARRNNEDAQITIYDRDYDISYSGCGIPYYVGGEVESRDDL

WP_031422658.1 --MKLVIIGSVAAGTSVAAKARRNNEDAQITIYDRDYDISYSGCGIPYYVGGEVESRDDL

WP_012727071.1 --MKLVIIGSVAAGTSVAAKARRNNEDAQITIYDRDYDISYSGCGIPYYVGGEVESRDDL

WP_016509108.1 --MKLVIIGSVAAGTSVAAKARRNNEDAQITIYDRDYDISYSGCGIPYYVGGEVESRDDL

WP_070328560.1 --MKLVIIGSVAAGTSVAAKARRNNEDAQITIYDRDYDISYSGCGIPYYVGGEVESRDDL

WP_071398091.1 --MKLVIIGSVAAGTSVAAKARRNNEDAQITIYDRDYDISYSGCGIPYYVGGEVESRDDL

WP_034817875.1 --MKLVIIGSVAAGTSVAAKARRNNEDAQITIYDRDYDISYSGCGIPYYVGGEVESRDDL

WP_034804577.1 --MKLVIIGSVAAGTSVAAKARRNNEDALITIYDRDYDISYSGCGIPYYVGGEVESRDDL

WP_047374565.1 --MKLVIIGSVAAGTSVAAKARRNNEDAQITIYDRDYDISYSGCGIPYYVGGEVESRDDL

WP_029334490.1 --MKLVIIGSVAAGTSVAAKARRNNEDAQITIYDRDYDISYSGCGIPYYVGGEVESRDDL

WP_024370588.1 --MKLVIIGSVAAGTSVAAKARRNNEDAQITIYDRDYDISYSGCGIPYYVGGEVESRDDL

WP_021066461.1 --MKLVIIGSVAAGTSVAAKARRNNEDAQITIYDRDYDISYSGCGIPYYVGGEVESRDDL

WP_029594929.1 --MKLVIIGSVAAGTSVAAKARRNNEDAQITIYDRDYDISYSGCGIPYYVGGEVESRDDL

WP_058764331.1 --MKLVIIGSVAAGTSVAAKARRNNEDAQITIYDRDYDISYSGCGIPYYVGGEVESRDDL

WP_034778796.1 --MKLVIIGSVAAGTSVAAKARRNNEDAQITIYDRDYDISYSGCGIPYYVGGEVESRDDL

WP_039810825.1 -MTKLLIIGSVAAGTSVGAKARRNSEDLQITIYDKDTDISYSGCGIPYYVGGEVADIDEL

WP_056061304.1 -MAKLLIIGSIAAGTSVGAKARRNSEDLEITIYDRDEDISYSGCGIPYFVGGEVAELDEL

WP_035408065.1 -MAKLLIIGSVAAGTSVGAKARRNSEDLEITIYDRDEDISYSGCGIPYFVGGEVAELDEL

WP_029333607.1 --MKLLIIGSVAAGTSVGAKARRNDESLQITIYDRDQDISYSGCGIPYFVGGEIEDIDTL

WP_053453049.1 --MKLMIIGSVAAGTSVGAKARRNSEDMQITIYDRDQDISYSGCGIPYFVGGEIADIDEL

WP_058705130.1 --MKLLIIGSVAAGTSVGAKARRNSEELQITIYDRDQDISYSGCGIPYFVGGEIADIDEL

WP_023467877.1 --MKLMIIGSVAAGTSVGAKARRNSEELQITIYDRDQDISYSGCGIPYFVGGEIADIDEL

WP_058713610.1 --MKLMIIGSVAAGTSVGAKARRNSEELQITIYDRDQDISYSGCGIPYFVGGEIADIDDL

WP_058265269.1 --MKLMIIGSVAAGTSVGAKARRNSEELQITIYDRDQDISYSSCGIPYFVGGEIADIDEL

WP_035397995.1 --MKLMIIGSVAAGTSVGAKARRNSEELQITIYDRDQDISYSGCGIPYFVGGEIADIDEL

WP_064300131.1 --MKLMIIGSVAAGTSVGAKARRNSEELQITIYDRDQDISYSGCGIPYFVGGEIADIDEL

WP_055967341.1 --MKLMIIGSVAAGTSVGAKARRNSEELQITIYDRDQDISYSGCGIPYFVGGEIADIDEL

WP_050677028.1 --MKLMIIGSVAAGTSVGAKARRNSEELQITIYDRDQDISYSGCGIPYFVGGEIADIDEL

WP_029341349.1 --MKLMIIGSVAAGTSVGAKARRNSEELQITIYDRDQDISYSGCGIPYFVGGEIADIDEL

WP_026831288.1 --MKLLIIGSVAAGTSVGAKARRNNEDLQITIYDRDQDISYSGCGIPYFVGGEIADIDEL

WP_014970324.1 --MKLLIIGSVAAGTSVGAKARRNNEDLQITIYDRDQDISYSGCGIPYFVGGEIADIDEL

WP_071500702.1 --MKLLIIGSVAAGTSVGAKARRNSEDLQITIYDRDQDISYSGCGIPYFVGGEIADIDEL

WP_028106360.1 --MKLLIIGSVAAGTSVGAKARRNSEDLQITIYDRDQDISYSGCGIPYFVGGEIADIDEL

WP_026833068.1 --MKLLIIGSVAAGTSVGAKARRNSEDLQITIYDRDQDISYSGCGIPYFVGGEIADIDEL

WP_026828434.1 --MKLLIIGSVAAGTSVGAKARRNSEDLQITIYDRDQDISYSGCGIPYFVGGEISDIDEL

WP_012370312.1 --MKLLIIGSVAAGTSVGAKARRNSEDLQITIYDRDQDISYSGCGIPYFVGGEIADIDEL

WP_071873852.1 --MKIVIIGSVAAGTSVAAKARRNTEEAEIVVYDQGKDISYSVCGIPYQIGGEVESIDEL

WP_073414130.1 --MKMIIIGSVAAGTSVAAKARRNDENIDITVYNADYDISYSICGIPYFLGGEVENLSSL

WP_066448669.1 --MKVIIIGSVAAGTSVAAKARRNDEEAEITLYNADYDISYSACGIPYFLGGEIENLDTL

WP_016201870.1 --MKVIIIGSVAAGTSVAAKARRNDETADITLYNADYDISYSICGIPYFLGGEVEELETL

WP_047943166.1 --MKVIIIGSVAAGTSVAAKARRNDEKAEITLYNADYDISYSICGIPYFLGGEVEELETL

WP_016204927.1 --MKVLIIGSVAAGTSVAAKARRNDENAEITLYNADYDISYSICGIPYFLGGEVENLETL

WP_053602939.1 --MKIIIIGSVAAGTSVAAKARRNDENAEITVYNADYDISYSICGIPYFLGGGVDELKAL

WP_010649726.1 --MKIIIIGSVAAGTSVAAKARRNDEQADITLYNADYDISYSICGIPYFLGGEVDTLETL

WP_074600543.1 --MKLIIIGSVAAGTSVAAKARRNDENAEITLYNADYDISYSICGIPYFLGGEVDELETL

WP_008633299.1 --MKIIIIGSVAAGTSVAAKARRNDENAEITLYNADYDISYSICGIPYFLGGEVEELDTL

WP_027953781.1 --MKIIIIGSVAAGTSVAAKARRNDENADITLYNADYDISYSICGIPYFLGGEVDELETL

WP_067725917.1 --MKIIIIGSVAAGTSVAAKARRNDENAEITLYNADYDISYSICGIPYFLGGEVDELESL

WP_071649283.1 --MKIIIIGSVAAGTSVAAKARRNDGNAEITLYNADYDISYSICGIPYFLGGEVDELETL

WP_010652163.1 --MKIIIIGSVAAGTSVAAKARRNDENAEIILYNADYDISYSICGIPYFLGGEVDELETL

WP_041844632.1 --MKIIIIGSVAAGTSVAAKARRNDEDAEITLYNADYDISYSICGIPYFLGGEVDELETL

WP_050739125.1 --MKIIVIGAVAAGTSAAAKARRNNDYADIVIYEKDQDISYSGCGLPYYIGGKVPSIESL

SEO96011.1 --MKLIVIGAVAAGTAAAAKARRNDDNADITIYEQDQDISYSSCGLPYYIGGEVEQIDEL

WP_069871897.1 --MRIVVIGAVAAGTSAAAKARRNDDQAEIVIYEKDQDISYSGCGLPYYIGGEIEAIEEL

OJV63954.1 --MRIVIIGAVAAGTSAAAKARRNDDQAEIVIYERDKDISYSGCGLPYYIGGEIEDIEEL

WP_066068147.1 --MKIIIIGAVAAGTSAAAKARRNNDSAKIVIYEKDGDISYSGCGLPYYIGGEIEDIEQL

WP_069999727.1 --MRILVIGAVAAGTSAAAKARRNNDKAEIVIYEKDQDISYSGCGLPYYIGGEIEDIGEL

SDP09337.1 --MRILVIGAVAAGTSAAAKARRNNDDAEIVIYERDNDISYSGCGLPYYIGGEIEDISEL

WP_023977032.1 --MRLIVIGAVAAGTSAAAKARRNDDNAEIVIYEKDRDISYSGCGLPYYIGKEIEDIGEL

SFD29588.1 --MRLLVIGAVAAGTSAAAKARRNDDDAEIVIYEKDRDISYSGCGLPYYIGKEIEDIGEL

KAJ53726.1 --MRILVIGAVAAGTSAAAKARRNDDTAEIVIYEKDKDISYSGCGLPYYIGGGIEDIGEL

WP_039633362.1 --MRILVIGAVAAGTSAAAKARRNNDKAEIIIYEKDKDISYSCCGLPYYIGGEIEDIGEL

WP_017472957.1 --MKLIVIGAVAAGTSAAAKARRNDDNAEIIVYEKDQDISYSGCGLPYYIGGEIEDIDAL

SFO84805.1 --MKLIVIGAVAAGTSAAAKARRNQDDLEITIYEKDVDISYSGCGLPYYIGNEVESIEEL

SFP14577.1 --MKLIVIGAVAAGTSAAAKARRNQDDLDITIYEKDIDISYSGCGLPYYIGNEVESIETL

WP_062323720.1 --MKLIVIGAVAAGTSAAAKARRNQDDLEITIYEKDVDISYSGCGLPYYIGNEVESIEAL

WP_073271217.1 --MRIIVIGAVAAGTSAAAKARRSDESAEITIYEKDEFISYSGCGMPYFIGGEIDNPEKL

WP_031573915.1 --MKIRIIGAVAAGTSAAAKARRNSEEAEIIIYEKDTYISYSGCGMPYYIGGEVENGEEL

WP_074913255.1 --MKIRIIGAVAAGTSAAAKARRNSEEAEIIIYEIDTYISYSGCGMPYYIGGEVENGEDL

OGO93293.1 --MRILIIGAVAAGTSAAAKARRNSEDAEIVLYEKDSYISYSGCGMPYYIGGELDNADEL

OJU15484.1 --MKIVIIGAVAAGTSAAAKARRNNEDAEIVMYEKDSFVSYSACGMPYYLGGTVESADEL

SCP98331.1 --MRIVIIGAVAAGTSAAAKARRNDETAEIVIYERDSYISYSGCGMPYYIGGEVENAREL

WP_066089511.1 --MRLIIIGAVAAGTSAAAKARRNDENAEILIYDKDSFISYSGCGMPYYIGGEVEDAQKL

SEW43766.1 --MRLVIIGAVAAGTSAAAKARRNDEDAQIVIYDKDNFISYSGCGMPYFIGGEVEDADEL

WP_014314980.1 --MRIVIIGAVAAGTSAAAKARRNSEDSEIVIYEKDNFISYSGCGMPYYIGGEVESAGEL

WP_033166250.1 --MRIIIIGAVAAGTSAAAKARRNSEEAEIIIYEKDNFISYSGCGMPYYIGGEIESADAL

WP_066648725.1 --MRIIIIGAVAAGTSVAAKARRNSESAEIVVYDKDSFISYSGCGMPYYIGGEVENADEL

WP_020072493.1 --MRIVIIGAVAAGTSAAAKAKRNNEDAEIVIYEKDSFISYSGCGMPYYIGDEIESADQL

WP_014314895.1 --MKIVIIGAVAAGTSAAAKARRNSEDAEIVIYEKDSFISYSGCGMPYYIGGEIDKADEL

WP_023388569.1 --MRILIIGAVAGGTSAAAKARRNSEDAEIIIFDKDSDISYSGCGLPYYIGGEVEKRSEL

WP_040428848.1 MYMKILIIGAVAAGTSAAAKARRNSEDAQIKIFEMDSDISYSGCGLPYYIGNEIEGRDKL

WP_073016597.1 --MKIAIIGAVAAGTSAAAKARRNDENAEIKIFEMGEEISYSSCGLPYYIGNEVQNREQL

WP_021801810.1 --MKIAIIGAVAAGTSAAAKARRNDETAEIKIFEMGEEISYSSCGLPYYIGNEIQNREQL

WP_018703748.1 --MKILIIGGVAAGTSAAAKARRNNETAEIKIFDQDEDMSYSVCGLPYYIGTEVKTREEL

WP_034869873.1 MNMKIVVIGAVAAGTSAAAKARRNDESAEIKIFDIDYNISYSGCGLPYYIGEQIEDRGEI

WP_073538592.1 --MKVVIIGAVAGGTSAAAKARRNDENTEIKIFDMDYDISYSGCGLPYYIGEEIQDRGDL

WP_007062926.1 MFMKVVIIGAVAGGTSAAAKVRRNDENVEIKIFDMDYDISYSGCGLPYYIGEEIGNRDEL

WP_066620912.1 --MKIVIIGAVAGGTSAAAKARRNDENAEIKIFDMDYDISYSGCGLPYYIGEEIQDREDL

WP_004455169.1 --MNILIIGAVAAGTSAAAKARRNDEKAEIKIYDMDYDISYSGCGLPYYIGEEIQDRDEL

WP_071167487.1 --MNILIIGAVAAGTSAAAKARRNDEKAEIKIYDMDYDISYSGCGLPYYIGEEIQDRDEL

WP_035776858.1 MYMKILIIGAVAGGTSAAAKARRNDENAEIKIYDMDYDISYSGCGLPYYIGEEIQDRDDL

WP_015613982.1 --MKIVIIGAVAGGTSAAAKARRNDEDAEIKIYDMDYDISYSGCGLPYYIGEEIQDRDEL

WP_026883985.1 --MKIVIIGAVAGGTSAAAKARRNDEEAEIIIFDKDKDISYSGCGLPYYIGNKVKDRGEL

WP_007930621.1 --MKIIIIGGVAAGTSAAAKARRNNENAQITLFDADSDVSYSGCGLPYFIGTEVEGREQL

WP_007952659.1 --MKIIIIGGVAAGTSAAAKARRNNENAQITLFDADSDVSYSGCGLPYFIGTEVEDREQL

.: :**.:*.**:..**.:*. * ::: . :*** **:** :. :

OHD09048.1 VPRNPEWFLKRHNIQIHTAHEVLKIDPSSKTLTVKNLSDQTVFEDSYDKLVLTTGASPIV

WP_038668288.1 NPRNPEWFKKRFAIDIKTEHEVIGIDADKKELGIKDLQTGTTFHDSYDILVLATGAAPVK

WP_026825315.1 TPRDAAFFKKRYNIDVMTRHNVTAVDKDAKTVTVENLDTGETFTDSYDVLVLATGATSIV

WP_074036424.1 TPRDAAFFKKRYNIDVMTRHNVTGVDKDAKTVTVENLETGDTFTDSYDVLVLATGATSII

WP_047794995.1 TPRDAAFFKKRYNIDVMTRHNVTAVDKDAKTVTVENLETGDTFTDAYDVLVLATGASSII

WP_074036076.1 TPRDAAFFKKRYNIDVMTRHNVTAVDKDAKTVTVENLETGVTFTDSYDVLVLATGASSII

WP_031422658.1 TPRDAAFFKKRYNIDVMTRHNVTAFDKDAKTVTVENLETGDTFTDSYDVLVLATGASSII

WP_012727071.1 TPRDAAFFKKRYNIDVMTRHNVTAVDKDAKTVTVENLETGDTFTDAYDVLVLATGASSII

WP_016509108.1 TPRDAAFFKKRYNIDVMTRHYVTAIDKDAKTVTVENLETGETFTDSYDVLVLATGASSIV

WP_070328560.1 TPRDAAFFKKRYNIDVMTRHNVTAIDKDVKTVTVENLETGETFTDSYDVLVLATGASSIV

WP_071398091.1 TPRDAAFFKKRYNIDVMTRHNVTAIDADAKTVTVENLETGETFTDSYDVLVLATGASSIV

WP_034817875.1 TPRDAAFFKKRYQLDVLTRHNVTAIDHEAQTLTVENLETGETFTDSYDRLVLATGASSII

WP_034804577.1 TPRDAAFFKKRYQLDVLTRHNVTAIDKDAQTIMVENLETGETFTDTYDRLVLATGASSII

WP_047374565.1 TPRDAAFFKKRYNIDVMTRHNVTAIDKDAQLVTVENLETGESFTDSYDKLVLATGASSIV

WP_029334490.1 TPRDAAFFKKRYQLDVLTRHNVTAIDKDAQTLTVENLETGETFTDTYDRLVLATGASSII

WP_024370588.1 TPRDAAFFKKRYQLDVLTRHNVTAIDKDAQTLTVENLETGETFTDTYDRLVLATGASSII

WP_021066461.1 TPRDAAFFKKRYNIDVITRHNVTAVDADAQTVTVENLETGESFTDSYDSLVLATGASSII

WP_029594929.1 TPRDAAFFKKRYNIDVMTRHNVTAVDADAQTVTVENLETGESFTDSYDSLVLSTGASSII

WP_058764331.1 TPRDAAFFKKRYQLDVMTRHNVTAIDADAQTITVENLETGESFTDSYDRLVLATGASSII

WP_034778796.1 TPRDAAFFKKRYQLDVMTRHNVTAIDADAQTITVENLETGESFTDSYDRLVLATGASSII

WP_039810825.1 TPRNAVWFKKRYNIDIHTQHEVLSINHEAKTAEILNLMTNETFTDSYDTLVLATGATSFV

WP_056061304.1 TPRDAAFFKKRYNIDVKTRHNVEAIDHETKTATIRHLDSSETFTDTYDTLVFATGASSII

WP_035408065.1 TPRDAAFFKKRYNIDVKTRHNVEAIDHETKTATIRHLDSGETFTDTYDTLVFATGASSII

WP_029333607.1 TPRDAAFFKKRYNIDVKTQHKVESINHQTKTATILNGVTGERFEDTYDTLVLATGASSII

WP_053453049.1 TPRDATFFKKRYNIDIHTQHEVESIDHATKTATIVNLLTGDCFTDTYDTLVLATGASSIV

WP_058705130.1 TPRDAAFFKKRYNIDIHTQHEVESIDHATKTATIVNLKTGDRFTDTYDTLVLATGASSIV

WP_023467877.1 TPRDAAFFKKRYNIDIHTQHEVESIDHVTKTATIVNLLTGDRFTDTYDTLVLATGASSIV

WP_058713610.1 TPRDAAFFKKRYNIDIHTQHEVESIDHATKTATIVNLLTGDRFTDTYDTLVLATGASSIV

WP_058265269.1 TPRDAAFFKKRYNIDIHTQHEVESIDHATKTATIVNLLTGDRFTDTYDTLVLATGASSIV

WP_035397995.1 TPRDAAFFKKRYNIDIHTQHEVESIDHATKTATIVNLLTGDRFTDTYDTLVLATGASSIV

WP_064300131.1 TPRDAAFFKKRYNIDIHTQHEVESIDHATKTATIVNLLTGNRFTDTYDTLVLATGASSII

WP_055967341.1 TPRDAAFFKKRYNIDIHTQHEVESIDHATKTATIVNLLTGDRFTDTYDTLVLATGASSII

WP_050677028.1 TPRDAAFFKKRYNIDIHTQHEVESIDHATKTATIVNLLTGDRFTDTYDTLVLATGASSII

WP_029341349.1 TPRDAAFFKKRYNIDIHTQHEVESIDHATKTATIVNLLTGDRFTDTYDTLVLATGASSII

WP_026831288.1 TPRDAAFFKKRYNIDIHTRHEVESIDHATKTATIVNLATGERLTDTYDTLVLATGASSIV

WP_014970324.1 TPRDAAFFKKRYNIDIHTQHEVESIDHATKTATIVNLATGERLTDTYDTLVLATGASSIV

WP_071500702.1 TPRDAAFFKKRYNIDIHTRHEVESIDHATKTATIINLTTGDKFTDTYDTLVLATGASSIV

WP_028106360.1 TPRDAAFFKKRYNIDIHTRHEVESIDHATKTATIVNLTTGDKFTDTYDTLVLATGASSIV

WP_026833068.1 TPRDAAFFKKRYNIDIHTRHEVESIDHATKTATIINLTTGDKFTDTYDTLVLATGASSIV

WP_026828434.1 TPRDAAFFKKRYNIDIHTRHEVESIDHATKTATIVNLATGDRLTDTYDTLVLATGASSII

WP_012370312.1 TPRDAAFFKKRYNIDIHTRHEVESIDHATKTATIVNLATGDRLTDTYDTLVLATGASSIV

WP_071873852.1 TPRNTAWFKKRYNVDIFTEHKVTKVNHETKTLEVLDVVTGETKEDSYDVLVLATGANPFT

WP_073414130.1 TPRSASWFKERYNVDIFTRHEVTQVNPAQKKIAVKDLETGKTKNDTYDVLVLATGASPTT

WP_066448669.1 TPRSASWFKKRYNVDIFTRHEVTTIDAEQKKVKVKNLDTDEVKEDSYDVLVFATGASPLT

WP_016201870.1 TPRSAAWFKKRYNVDIFIRHEVIKIDAKQKKIQVKNLDTNEIKEDTYDVLVFATGASPIT

WP_047943166.1 TPRSAAWFKKRYNVDIFTRHEVTTIDAKQKKVQVKNLDTNEVKEDTYDVLVFATGASPIT

WP_016204927.1 TPRSAAWFKKRYNVDIFTRHEVTTIDAEQKKVQVKNLDTNEIKEDSYDVLVFATGASPIK

WP_053602939.1 TPRNAPWFKKRFNVDIFTRHEVTKVDAEQKKVTVKNLDTDEIKEDSYDTLVFATGATPIT

WP_010649726.1 TPRSALWFKKRYNVDIHTRHEVTEIDPEQKRVTVKNLNNNEVMEDTYDTLVFATGATPIT

WP_074600543.1 TPRNAAWFKKRYNVDIHTRHEVTAIEPNQKTVTVKNLETGEEIQDNYDTLVFATGASPIT

WP_008633299.1 TPRSAAWFKKRYNVEIFTRHEVTRINSETKKVVVKKLDTGETIEDDYDTLVFATGASPNR

WP_027953781.1 TPRSSEWFKKRYNVDIHTRHEVVEINPDQKVVTVKNLNTNEIIEDHYDTLVFATGAAPTT

WP_067725917.1 TPRSAAWFKKRYNVNILTRHEVTTIDPESKKVIVKNLDTNETKEDHYDTLVFATGATPIT

WP_071649283.1 TPRSAAWFKKRYNVDIQTRHEVIKIDPDKKVITVKNLDTSETKEDSYDTLVFATGATPIT

WP_010652163.1 TPRSAEWFKVRYNVDIHTRHEVTTIDPDKKSVTVKNLDTNETKEDHYDTLVFATGATPIT

WP_041844632.1 TPRSAAWFKVRYNVDIHTRHEVTSINPEKKTVIVKNLDTNETKEDHYDTLVFATGATPIT

WP_050739125.1 VPRDAGFFKAKYNIDIFTRHEVLKIDIYHKTLLVKNLVTGNTFKDVYDKLIISTGAHAFV

SEO96011.1 TPRDAHFFKKKYNINVCTGHQVQQINYRVKTITVKNLATEEVFDDSFDKLVIATGASPVF

WP_069871897.1 TPRDARFFKKKYDIEVKTLHEVLEIYPEQKSLLVKDLSTGSVFADTYDKLVFATGAIPFV

OJV63954.1 TPRDTKFFKKKYNIEVKTEHEVMEIFPDQKSVLVKDIATGKLFGDTYDKLVIATGAMPFV

WP_066068147.1 TPRDAQFFKKKYNIDIFTRHEVLKIDTKSKKVIVKNLSTDEIFEDNYDKLVIATGASPFI

WP_069999727.1 TPRDPLFFRKKYNIDIHTGHEVIKINPEEKKLVIKNIQTNEAFEDYYDKLVIATGATPFV

SDP09337.1 TPRDSKFFKKKYNVDVLTGHEVLNINPNTKELTVKNLNTQEIFVDKYDKLILGTGATPFM

WP_023977032.1 TPRDSAFFKKKYNIDVFTGYEVLNINPDLKEVEVKNLNTNEVFKDKYDKLIIATGASPFI

SFD29588.1 TPRDSSFFKKKYNIDVFTGHEVLNINSDLKEVSVLNLSTNEVFKDNYDKLIIATGASPFV

KAJ53726.1 APRDSLFFKNKYNIDIFTGFQVLNINPELKGLKVKNLYTDEIFEDKYDKLIIATGAIPFV

WP_039633362.1 IPRDSIFFKKKYNIDIFTGHEVLDINPQTKEITIKNLHTNKVFIDNYDKLIIATGAEFFI

WP_017472957.1 TPRDPIFFKKKYNIDIFTRHEVVAVDPSEKRVTVKNLATGEQFNDAYDKLVIATGASPVM

SFO84805.1 TPRDPAFFKKKYNIDIHTAHLVTKIDHDNQQVYVKNLQTDETFTDSYDKLVIATGAKSIV

SFP14577.1 TPRDPAFFKKKYNIDIHTAHLVTEIDHDNQQVHVKNLKTGEIFTDAYDKLVIATGAKSIA

WP_062323720.1 TPRDPAFFKKKYNIDIHTAHLVTKIDHDNQQVHVKSLKTNETFTDAYDKLVIATGAKSIV

WP_073271217.1 TPRDPAFFKSKYNIDVLTKHEVLYLNGENKTVKVRNLVTGEEFEDCYDKLVLATGAYSVK

WP_031573915.1 TPRDPKFFKSKYNVDIKTGYEVLHVDAARKILKVQNLETSEVFEDTYDKLILATGARSVV

WP_074913255.1 TPRDPKFFKSKYNVDIKTGHEVLQVDPKEKILLVKNLETGETMKDSYDKLIFATGARSVV

OGO93293.1 TPRDPAFFKSKYNIDILTQHEVLSIHPNMKTIRVKNLTNDEQFIDTYDKLIIATGARAVM

OJU15484.1 TPRDPAFFHRKYNVTIKILHKVLAIHPETKTLIVKNLLTGEVFSDIYDTLVIATGAKPVI

SCP98331.1 TPRNSAFFKSKYNVDILTGHEVLSIDAKNKILEIRNLSTGEILKDSYDKLVIATGASAVL

WP_066089511.1 TPRDSAFFKSKYNVDIFISHEVLSINHINKTLEIKNLLTGNIFTDHYDKLIIATGASAVI

SEW43766.1 TPRDPAFFKSKYNVDVFILHEVLSINVHEKSMKIKNLSTGEEFEDTYDKLVIATGARAIL

WP_014314980.1 TPRDPAFFKSKYNVDIHTLHEVVSILPDEKKVSVRNLSTDEVFTDYYDRLVLATGARAVI

WP_033166250.1 TPRDPAFFKSKYNVDIYTLHGVLSIHPEEKKIEIRNLLTDEVFNDNYDKLVLATGARAVI

WP_066648725.1 TPRDPAFFKSKYNVDILIRHEVLSINPDQKVLKVKNLTTGEVFSDSYDKLVIATGAQAVV

WP_020072493.1 TPRSPAFFKSKYNVDIHTRHEVLSISPDVKTITIKNLITEDIFNDHYDKLVIATGARATI

WP_014314895.1 IPRDPAFFKKKYNVDIFIRHEVLAISPDSRNITVKNLTTGEMITDHYDKLVFATGARATV

WP_023388569.1 VPRDAAFFRDKYNVDVRTSHEVLAIDPTRKSLSVRDLTDGREYEESYDKLIIATGARPFV

WP_040428848.1 VPRNAAFFKSKYNVDILTKHKVLGINTDEKSLKVINLETSEVFVEKYDKLVIATGARPIL

WP_073016597.1 VPRDVKFFKSKYNVDILIRHKVLSIDTKAKELMVEDLTNGKVFIEKYDKLVISTGATPIF

WP_021801810.1 VPRDVKFFKSKYNVDILIRHKVLSVDVNAKELKVENLSSGKVFIEKYDKLVISTGATPIL

WP_018703748.1 VPRDAAFFKKKYNVDVYMGHQAVAIDIANKVIIVKNLRSQEQFEEAYDRLILATGASVRI

WP_034869873.1 VPRDAGFFKKKYNIDIFIRHEVLKINIKDKVLIVRNLDTSKVFNESYDKLIIATGAKPVV

WP_073538592.1 VPRNAEFFKKKYNVDVFRRHEVLKIITSYKVLIVKNLETNEIFTENYDKLVIATGAKPIV

WP_007062926.1 TPRNAEFFKKKYNVDVFRRHEVLKINSEDKVLTIKNLDTNNVFNEKYDKLVIASGARSII

WP_066620912.1 TPRNAEFFKKKYNVDVFRRHEVLNINTEDKALTVKNLDTNDVFTESYDKLVIATGAKSIV

WP_004455169.1 TPRNAEFFKKKYNVDVFRRHEVLKINTDQKILTIKNLDTNNVFDERYDKLVIATGAKPIV

WP_071167487.1 TPRNAEFFKKKYNVDVFRRHEVLKINTDQKILTIKNLDTNNVFDERYDKLVIATGAKPIV

WP_035776858.1 TPRNAEFFKKKYNVDVFRRHEVLKIDTEHKTLNIKNLDTNNVFDESYDKLVIATGAKPIV

WP_015613982.1 TPRNAAFFKKKYNVDVFRRHEVLKINTENKVLTIKNLDTDDVFDESYDKLVIATGAKPIV

WP_026883985.1 VPRDANFFKKKYNVDVFTQHEVLSIDASGKKLTVKNLNTEEVFVEEFDKLVVSTGARSVI

WP_007930621.1 VPRDAAFFKQKYNVDVYTRHQVLEIQPQSKVLTVQNLVTQEIFQERYDKLVVATGAKSVV

WP_007952659.1 VPRDAAFFKQKYNVDVYTRHQVLEIQPQSKVLTVQNLVTQEIFQERYDKLVVATGAKSVV

**. :* :. : : . . . : : : :* *:. :**

OHD09048.1 -PDIKNIHSPHVFFLRNVKDADALKSFILTNKPKNALIIGSGFIALEMLESLSNIGINTT

WP_038668288.1 -PDIKGLEGNNVFIVRNITSADKIKEHIEKEHPASAVIIGSGFIGMEIAENLVKKGIQIA

WP_026825315.1 -PPIPGVKSDNVFPIRNIQHAEATRSFVDSTDPKHATIIGAGFIGLEMAEQLKLRGVEVT

WP_074036424.1 -PPIPGVKSDNVFPIRNIQHAEATRSFVDSTNPKHATIIGAGFIGLEMAEQLKLRGVDVT

WP_047794995.1 -PPIPGVKSDNVFPIRNIQHAEATRSFVDSTDPKHATIIGAGFIGLEMAEQLKLRGVDVT

WP_074036076.1 -PPIPGVKSDNVFPIRNIQHAEATRSFVDSTDPKHATIIGAGFIGLEMAEQLKLRGVDVT

WP_031422658.1 -PPIPGGKSDNVFPIRNIQHAEATRSFVDSTDPKHATIIGAGFIGLEMAEQLKLRGVDVT

WP_012727071.1 -PPIPGVKSDNVFPIRNIQHAEATRSFVDSTDPKHATIIGAGFIGLEMAEQLKLRGVDVT

WP_016509108.1 -PPIPGVKSDNVFPIRNIQNAEATRNFVDATNPKHATVIGAGFIGLEMAEQLKLRGIDVT

WP_070328560.1 -PPIPGVKSDNVFPIRNIQNAEATRNFVDATNPKHATVIGAGFIGLEMAEQLKLRGIDVT

WP_071398091.1 -PPIPGVKSDNVFPIRNIQNAEATRNFVDATNPKHATVIGAGFIGLEMAEQLKLRGIDVT

WP_034817875.1 -PPIPGVDSDNVFPIRNIQNAETTRNFVDATSPKHATIIGAGFIGLEMAEQLKLRGVDVT

WP_034804577.1 -PPIPGVDSDNVFPIRNIQNAETTRHFVDATSPKHATIIGAGFIGLEMAEQLKLRGVDVT

WP_047374565.1 -PPIPGVDSDNVFPIRNIQNAEATRNFVDATNPKHATIIGAGFIGLEMAEQLKLRGVDVT

WP_029334490.1 -PPIPGVDSDNVFPIRNIQNAEATRNFVDATNPKHATIIGAGFIGLEMAEQLKLRGVDVT

WP_024370588.1 -PPIPGVDSDNVFPIRNIQNAEATRNFVDATNPKHATIIGAGFIGLEMAEQLKLRGVDVT

WP_021066461.1 -PPIPGVDSDNVFPIRNIQNAEATRNFVDATNPNHATIIGAGFIGLEMAEQLKLRGVEVT

WP_029594929.1 -PPIPGVDSDNVFPIRNIQNAEATRNFVDATNPKHATIIGAGFIGLEMAEQLKLRGVEVT

WP_058764331.1 -PPIPGVDSDNVFPIRNIQNAEATRNFVDATNPKHATIIGAGFIGLEMAEQLKLRGIEVT

WP_034778796.1 -PPIPGVDSDNVFPIRNIQNAEATRNFVDATNPKHATIIGAGFIGLEMAEQLKLRGIDVT

WP_039810825.1 -PPIPGVEADNVFSVRNIRNAGEIRTYIDQHQPKHVTIVGGGFIGLEMAEQLKYKGLEVT

WP_056061304.1 -PPLPGVDLENVFPVRNIRNADAIRRYVDAQAPKRAVIVGGGFIGLEMAEQLTYRGIEVT

WP_035408065.1 -PPLPGVDLENVFPVRNIRNAEAIRRYVDAQAPKRAVIVGGGFIGLEMAEQLTYRGIEVT

WP_029333607.1 -PPFPGVDKRNVFSVRNVQNAGAIRSYIEQHDPKTVTIVGGGFIGLEMAEQLTYRGITVT

WP_053453049.1 -PPLPGVEQENVFTVRNVRNADAIRSYIDAHDPKTATIVGGGFIGLEMAEQLTYRGIQVT

WP_058705130.1 -PPLPGIEHENVFTVRNVRNADAIRSYIDANDPKTATIVGGGFIGLEMAEQLTYRGIQVT

WP_023467877.1 -PPLSGVEHENVFTVRNVRNADAIRSYIDAHDPKTATIVGGGFIGLEMAEQLTYRGIQVT

WP_058713610.1 -PPLSGVEHENVFTVRNVRNADAIRSYIDAHDPKTATIVGGGFIGLEMAEQLTYRGIQVT

WP_058265269.1 -PPLPGVEHENVFTVRNVRNADAIRSYIDAHDPKTATIVGGGFIGLEMAEQLTYRGIQVT

WP_035397995.1 -PPLPGVEHENVFTVRNVRNADAIRSYIDAHDPKTATIVGGGFIGLEMAEQLTYRGIQVT

WP_064300131.1 -PPLPGVEHENVFTVRNVRNADAIRSYIDAHDPKTATIVGGGFIGLEMAEQLTYRGIQVT

WP_055967341.1 -PPLPGVEHENVFTVRNVRNADAIRSYIDAHDPKTATIVGGGFIGLEMAEQLTYRGIQVT

WP_050677028.1 -PPLPGVEHENVFTVRNVRNADAIRSYIDAHDPKTATIVGGGFIGLEMAEQLTYRGIQVT

WP_029341349.1 -PPLPGVEHENVFTVRNVRNADAIRSYIDAHDPKTATIVGGGFIGLEMAEQLTYRGIQVT

WP_026831288.1 -PPFPGVDHDNVFTVRNVRNADAIRSYIDAHDPKTATIVGGGFIGLEMAEQLTYRGIQVT

WP_014970324.1 -PPFPGVDHDNVFTVRNVRNADAIRSYIDAHDPKTATIVGGGFIGLEMAEQLTYRGIQVT

WP_071500702.1 -PPFPGVDHDNVFTVRNVRNADAIRSYIETHDPKTATIVGGGFIGLEMAEQLTYRGIQVT

WP_028106360.1 -PPLQGVDHDNVFTVRNVRNADAIRSYIDAHDPKTATIVGGGFIGLEMAEQLTYRGIQVT

WP_026833068.1 -PPLPGVDHDNVFTVRNVRNADAIRSYIDAHEPKTATIVGGGFIGLEMAEQLTYRGIQVT

WP_026828434.1 -PPFPGVDHDNVFTVRNVRNADAIRSYIEQHDPKTATIVGGGFIGLEMAEQLTYRGIQVT

WP_012370312.1 -PPLQGVDHDNVFTVRNVRNADAIRSYIDAHNPKTATIVGGGFIGLEMAEQLSYRGIQVT

WP_071873852.1 PPPFNQGAYNNVFQVRNIQDNRDITAYLSLKQPKKAVVVGSGFIGLEMTEQLVHKGMKVT

WP_073414130.1 -PNIPGVNKEHVFQVRTIQNTLKIDQYMKKENSQKATIIGAGYIGLEIAEQLIHKGLEVT

WP_066448669.1 -PDVSGVDKEHVFQVRTIQHTAAIDQFIKINQPKKATIIGAGYIGLEMAEQLTQRGLEVT

WP_016201870.1 -PNIPGVEEDHVFQVRTIQNTVAIDQYMKANQPKKATIIGAGYIGLEMAEQLSHRGLDVT

WP_047943166.1 -PAISGVDGEHVFQVRTIQNTAAIDQYMKENQPKKATIIGAGYIGLEMAEQLTHRGLDVT

WP_016204927.1 -PDIDGVDGEHVFQVRTIQNTAAIDQYMKANQPKKATIIGAGYIGLEMAEQLTHRGLEVT

WP_053602939.1 -PNIPGVDKEHVFHVRTIQNTFAIDRYMQKNEPKKATIVGAGYIGLEMAEQLTQKGLEVT

WP_010649726.1 -PSIPGVEKNHVFQIRTIQNTTQVDEYLKINSPKKVTIIGAGFIGLEMAEQLIQKGLEVT

WP_074600543.1 -PQIDGVELGHVFHVRTIQNTAAIQSFMTVNQPKKVTIIGAGFIGLEMAEQLTHKGMEVT

WP_008633299.1 -PNIEGVGHDHVFQVRTIQNTAMIDSFMKANHPKKVTIIGAGFIGLEMAEQLRHKGLDVT

WP_027953781.1 -PQIDGVEMEHVFHVRNIQNTASIHSYMQAESPEKVTIIGAGFIGLEMAEQLKHRGLDVT

WP_067725917.1 -PTIDGVHQEHVFQVRTIQHTASIDSYMRANNPQKVTIIGAGFIGLEMAEQLTHKGLEVT

WP_071649283.1 -PPIKGVEMEHVFHVRNIQNTASINSFMVSNNPKKVTIIGAGFISLEMAEQLTHKGLEVT

WP_010652163.1 -PKIVGVDMDHVFHVRNIQNTAAINNFMGTNNPKKVTIIGAGFIGLEMAEQLTLKGLEVT

WP_041844632.1 -PKIDGVDRKHVFHVRTIQNTAAINEFMNLNNPKKVTIIGAGFIGLEMAEQLTHKGLDVT

WP_050739125.1 -PPIKGIHNANVFFLRNVQNAVAIKTFMQKHLPKTAVIVGSGFIGFEVMENLIDAGIKVI

SEO96011.1 -PKIRGIDQKHVFGLRTVEHARQIKNYLQQQQPKTAVIVGTGFIGFEMLENLHELGIDVT

WP_069871897.1 -PRVEGIDSEHVFCLRNVQNAIAIKHFIDVKKPKHAVIAGTGFIGFEVLENLMHQNIEVT

OJV63954.1 -PSIQGVDLEHVFFLRNVQNAIAIKKFIDEKKPEHAVIAGTGFIGFEVLENLMHLNIDTT

WP_066068147.1 -PDIKGVDKEHVFFLRNVKHAQNIKNFIDSKHPKHAVIAGTGFIGFEMLENLILRNIDVT

WP_069999727.1 -PHVNARGNQHVFFLRNVQSARSIRDFIEVQKPQRALIAGTGFIGFEMLENLMAEGIDVT

SDP09337.1 -PNIKGIKNKNVFFLRNVQSARNIRGFIENNKPKHAVIVGTGFIGFEMLENLMDTGVNVT

WP_023977032.1 -PKIEGIDNNNVFFLRNVQSAKNIRNFIDNKKPKHAVIAGTGFIGFEMLENLMSDGVNVT

SFD29588.1 -PPIEGIDKNNVFFLRNVQSARNIRDYIDNKKPKNAVIVGTGFIGFEMLENLICDGINVT

KAJ53726.1 -PNVKGIDNNNVFFLRNVESAKNIRNFIEERKPQYAVIAGTGFIGFEMLENLMTDGIKVT

WP_039633362.1 -PNVKGIDRGNVFFLRNVQSAKNIKNFIEEKKPQHAIIAGTGFIGFEMLENLMSDGINVT

WP_017472957.1 -PPIEGVDRDHVFALRHVQHARDIRRFIIDKQPKSAVIAGTGFIGFEMLENLRAEGIDVT

SFO84805.1 -PPIDGVDLPHVHSLRTVQDARRIKGYIDEHQPKKAVVVGTGFIGFEMLENLIERDIDVV

SFP14577.1 -PPIDGVDLPHVHALRTVQDARQIKGYIDDHQPKKAVIVGTGFIGFEMLENLIERDIEVV

WP_062323720.1 -PPIDGVDLPHVHALRTVQDARQIKGYIDEHQPKTAVVVGTGFIGFEMLENLIERDIEVV

WP_073271217.1 -PPIEGMDKNNTFSLRNITDMYRIKEYLEESAPSKATIVGSGFIGLELCESFKALGLEVA

WP_031573915.1 -PPIPGVENKHVFSLRNINDMYRIKEYIENNSPKQAVTIGTGFIGLEMAENLKHLSMDVT

WP_074913255.1 -PPITGVRNKHVFSLRNIKDMYSIKKYIDEKNPKDAVIIGTGFIGLEMAENLKHLGMEVT

OGO93293.1 -PPVPGVDKSHVFTLRSINDMNRIKAFIDNNKPGTAAIIGTGFIGLEMSENLKRLGISIT

OJU15484.1 -PPIKGAEHKHVYALRTIGDMNRIKAYLDENAPKSAAIIGTGFIGLEVCENLNKIGLEVT

SCP98331.1 -PPIQGVEADNVFTLRNIGDMNRIKAYLNEHAPKKAVIIGTGFIGLEVCENLKGLGIDVT

WP_066089511.1 -PPVKGAELKNVFTLRNIGDMNQIKGYINEFSPKSAVIVGTGFIGLEVCESLREIGMDVT

SEW43766.1 -PPIKGAENKNAFTLRNIGDMKKIKSYINEFLPKTAVIIGTGFIGLEVCENLKKLGINVT

WP_014314980.1 -PPIKGTDFEHVFTLRNINDMNQIKNFIDSHRPQSAVIVGTGFIGLEVCENLQKLGINVT

WP_033166250.1 -PPIKGTDFKHVFSLRNINDMNRIKEYINSYHPQSTVIVGTGFIGLEVCENLKSLGINIT

WP_066648725.1 -PPIKGADGRHVFTLRNINDMNRIKVYIDENRPKRAAIIGTGFIGLEVCENLKKLGLEAT

WP_020072493.1 -PPIKGADSRHVFTLRNINDMNHIKAFIDTNRPQSAAIIGTGFIGLEVCENLKGLGIEVT

WP_014314895.1 -PPIKGADSQHVFTLRNINDMNRIKDFIGTNCPKSAAIIGTGFIGLEVCENLKGLGMDVT

WP_023388569.1 -PQMEGISLDGVFTLRNVKSADRIREFLRSRSPKRAVIVGTGFIGLELCEVLVKNGLEVH

WP_040428848.1 -PPIKGIDKANVFTLRNVESADNIKSYIQNNNPKRALVIGSGFIGLEMVENLENLGIEVS

WP_073016597.1 -PRIEGIDKDNVFVLRDVISADKIKNYIEKSKPQKALVVGSGFIGLEMAENLKNIGIDVT

WP_021801810.1 -PRIEGIDKDNVFVLRDVISADKIKNYIEKSKPKKALVVGSGFIGLEMVENLKNIGIDVT

WP_018703748.1 -PSIPGIEKENVFSLRNVRSADRIRAYVLEQQPQRAVVVGSGFIGLEVAENLKARGLEVT

WP_034869873.1 -PNIKGADLENIFYLRNVQQADKIKQYILVNNPKKAVIVGTGFIGLEMAENLKDLGIEVT

WP_073538592.1 -PNIPGAEKSNVFYLRNVLQADKIKEYILAHKPKSAVIIGTGFIGLEMAENLKNLGIEVT

WP_007062926.1 -LNIPGVEKSNVFYLRNVKQADKIKEYIMKNKPKSVVIVGTGFIGLEMTENLKNIGIKVT

WP_066620912.1 -PNIPGAENSNVFYLRNVTQADKIKEYIITHKPKSAVIVGTGFIGLEMTENLKNLGIEIT

WP_004455169.1 -PDIKGAEKSNVFYLRNVMQADKIKEYILSHKPKSAVIVGTGFIGLEMTENLKRLGIQVT

WP_071167487.1 -PDIKGAEKSNVFYLRNVMQADKIKEYILSHKPKSAVIVGTGFIGLEMTENLKRLGIQVT

WP_035776858.1 -PNIEGAEKSNVFYLRNVRQADKIKEYILNYKPKSAVIIGTGFIGVEMTENLKKLGIEVT

WP_015613982.1 -PNIEGAEKSNVFYLRNVRQADKIKEYILSHKPKSAVIVGTGFIGLEMTENLKKLGIEVT

WP_026883985.1 -PNIPGVDKNNVFYLRNVQSADRIKNFINENNPKKVVIVGSGFIGLEMTENLKELGIDVT

WP_007930621.1 -PAIEGGNKPNVFYLRNVINADRIREQVLTANPKSAVIVGSGFIGLEMAENLTARGIAVS

WP_007952659.1 -PAIEGGNKPNVFYLRNVINADRIREQVLTANPKSAVIVGSGFIGLEMAENLTARGIAVS

. . :* : : . . * *:*..*: * : .:

OHD09048.1 VIERENRLMPRLDPDISILLENELEKRGIRFVLSQSVNEIHQK----LVSTDKGSVYEAD

WP_038668288.1 VVEALGQVMPSYDPEMSLRIKKELIENKVQVFSGEKVVKVDDENK--SVLTDTGRMLPAD

WP_026825315.1 IIERVPQVMPPLDKDMACRVEEHLEQNGIELMLGETVTELIGDGHVERVVTESGKTIETD

WP_074036424.1 IIERVPQVMPPLDKDMACRVEEHLEKNGIELMLGETVTELVGDGHVERVVMESGKTIETD

WP_047794995.1 IIERVPQVMPPLDKDMACRVEEHLEKNGIELMLGETVTELVGDGHVERVVTESGKTIETD

WP_074036076.1 IIERVPQVMPPLDKDMACRVEEHLEKNGIELMLGETVTELVGDGHVERVVTESGKTIETD

WP_031422658.1 IIERVPQVMPPLDKDMACRVEEHLEKNGIELMLGETVTELVGDGHVERVVTESGKTIETD

WP_012727071.1 IIERVPQVMPPLDKDMACRVEEHLEKNGIELMLGETVTELVGDGHVERVVTESGKTIETD

WP_016509108.1 IIERIPQVMPPLDKDMACRVEDHLEKNGIELLLGETVTELVGDGHVERVVTESGKTVETD

WP_070328560.1 IIERIPQVMPPLDKDMACRVEDHLEKNGIELLLGETVTELVGDGHVERVVTESGKTIETD

WP_071398091.1 IIERIPQVMPPLDKDMACRVEDHLEKNGIELLLGETVTELVGDGHVERVVTESGKTIDTD

WP_034817875.1 VIERIPQVMPPLDKDMACRVEDHLEKNGIELLLGETVTELVGDGHVERVVTESGKTIDTD

WP_034804577.1 VIERIPQVMPPLDKDMACRVEDHLEKNGIELLLGETVTELVGDGHVERVVTKSGKTIDTD

WP_047374565.1 VIERIPQVMPPLDKDMACRVEEHLEKNGIELLLGETVTELVGEGHVERVVTESGKTIDTD

WP_029334490.1 VIERIPQVMPPLDKDMACRVEEHLEKNGIELLLGETVTELVGEGHVERVVTESGKTIDTD

WP_024370588.1 VIERIPQVMPPLDKDMACRVEEHLEKNGIELLLGETVTELVGEGHVERVVTESGKTIDTD

WP_021066461.1 VIERFPQVMPPLDKDMACRVEDHLEKNGIELMLGETVTELVGDGHVERVVTESGKTIDTD

WP_029594929.1 VIERFPQVMPPLDKDMACRVEDHLEKNGIELMLGETVTELVGDGHVERVVTESGKTIDTD

WP_058764331.1 VIERFPQVMPPLDKDMACRVEGHLEKNGIELMLGETVTELIGDGHVERVVTESGKTIDTD

WP_034778796.1 IIERFPQVMPPLDKDMACRVEDHLEKNGIELMLGETVTELIGDGHVERVVTESGKTIDTD

WP_039810825.1 LIERLPQVMPPLDLDMAERVARHLQEKGVKLLTGQTVQSLYDGERVSTVQLESGQTIETD

WP_056061304.1 LVERLAQVMPPLDVDMAKRVSDHLEANGVILRLGESVASFEGEKTARAVVLASGETIETD

WP_035408065.1 LVERLTQVMPPLDVDMAKRVSDHLEANGVILRLGESVTSFEGEKTARTVVLASGETIETD

WP_029333607.1 LVERLPQVMPPLDFDMASRVGDHLESKGVRLLLNESVTALTGEGDVEQVELASGKSIAAD

WP_053453049.1 LVERLPQVMPPLDRDMAERVADHLKDKGVSLLLGESVTAFNGKEGVSEVALESGKTIETD

WP_058705130.1 LVERLPQVMPPLDRDMAERVADHLKDKGVSLLLGESVTAFNGEERISEVALESGKTIETD

WP_023467877.1 LVERLPQVMPPLDRDMAERVADHLKDKGVSLLLGESVTAFNGEARVSEVALESGKTIETD

WP_058713610.1 LVERLPQVMPPLDRDMAERVADHLKDKGVSLLLGESVTAFNGEERVSEVALESGKTIETD

WP_058265269.1 LVERLPQVMPPLDRDMAERVADHLKDKGVSLLLGESVTAFNGEERVSEVALESGKTIETD

WP_035397995.1 LVERLPQVMPPLDRDMAERVADHLKDKGVSLLLGESVTAFNGEERVSEVALESGKTIETD

WP_064300131.1 LVERLPQVMPPLDRDMAERVADHLKDKGVSLLLGESVTAFNGEERVSEVALESGKTIETD

WP_055967341.1 LVERLPQVMPPLDRDMAERVADHLKDKGVSLLLGESVTAFNGEERISEVALESGKTIETD

WP_050677028.1 LVERLPQVMPPLDRDMAERVADHLKDKGVSLLLGESVTAFNGEERISEVALESGKTIETD

WP_029341349.1 LVERLPQVMPPLDRDMAERVADHLKDKGVSLLLGESVTAFNGEERISEVALESGKTIETD

WP_026831288.1 LVERLPQVMPPLDRDMAERVADHLRDKGVSLLLGESVTAFNGESRVTEVALESGKSIETD

WP_014970324.1 LVERLPQVMPPLDRDMAERVADHLRDKGVSLLLGESVTAFNGESRVTEVALESGKSIETD

WP_071500702.1 LVERLPQVMPPLDRDMAERVADHLRDKGVSLLLGESVTALNGAERVTDIALESGKSIETD

WP_028106360.1 LVERLPQVMPPLDRDMAERVADHLRDKGVSLLLGESVTAFNGTERVTEVALESGKSIETD

WP_026833068.1 LVERLPQVMPPLDRDMAERVADHLRDKGVSLLLGESVTAFNGTERVTEVALESGKSIETD

WP_026828434.1 LVERLPQVMPPLDRDMAERVADHIRDKGVSLLLGESVTALNGTERVTDIALESGESIETE

WP_012370312.1 LVERLPQVMPPLDRDMAERVADHLRDKGVSLLLGESVTALNGTERVTDIALESGKSIATE

WP_071873852.1 LVEMQDQVMPPMDADMAFRVEEHLRAHGVRLLLSDTVKTIDGESSVKSVTTTKGETLEAD

WP_073414130.1 MIQRSNQMMPNLDKDMAFRVEEELRKHGVQLLLNEEAILIEDN----SVTTKSGQSIQSD

WP_066448669.1 IVQRSNQVMAHLDKDMACRVEEHLVKKKVNLILNDEVTEISKK----EVLTKSGETIATD

WP_016201870.1 IIQRSNHVMTHMDKDIASRVEEHLVKKGITLILNEEVTVIDKM----NVQTKSGKFIGTD

WP_047943166.1 IVQRSNHVMAHMDKDIASRVEEHIVKKGVNLILNDEVTAISQK----EVQTKSGKAIETD

WP_016204927.1 IVQRSNHVMAHMDKDIASRVEEHLVKKGVNLILNDEVTAISKK----EVQTKSGKAIETD

WP_053602939.1 IIQRSNQVMPHFDKDMAFRVEEELLKHGVNLLLNEEASYISDN----TVETKSGKVIESD

WP_010649726.1 IVQRSNQVMPHFDKDMAFRVEEHLEQKGVNLLLNEEVNAISDN----RVETKSGTVIEAD

WP_074600543.1 IVQRGGQIMSHLDRDMAVRVEEHMRNYGVNLLVNEEAAKISPH----RVEMASGKAVDAD

WP_008633299.1 IVQRSGQIMPHLDLDMARRVETHIRDHGVTLLLNEEVHTISKT----TLETKSGTTLESD

WP_027953781.1 IVQRSNQIMPHLDKDMAFRVEEHLRANGVNLLLNEEAAAISET----SVETTKGSVIDAD

WP_067725917.1 LIQRSDQVMPHLDKDMAKRVEEHVRNNGVNLLLHEEASTISEN----SVETKSGKAIESD

WP_071649283.1 IVQRSNQIMPHLDKDMAFRVEEHIRDNGVNLLLNEEATVITEN----TVETKSGNVIDSD

WP_010652163.1 IVQRSNQIMPHLDKDMAFRVEEHLRDSGVDLLLNEEASKISEQ----TVETKNGKVIDTD

WP_041844632.1 IVQRSNQIMPHLDKDMAFRVEEHLRNNGVKLLLNEEASVITEK----AVETKSGKMIDSE

WP_050739125.1 LVERAGKLTPNLDEDMSKYLESILSKMEISIFKNSNVVEATNQ----GVLLDNGTLIPGE

SEO96011.1 IVEKNSQITPNLDEDMAQYLETQLRAKGIKIIKQAELEEINSD----HVVLSQHGKLSAE

WP_069871897.1 IVEVANKITPNLDQDMAMFLEKTLVQKGIKIIKSQSIAKIEAE----QITLNGGNTLKAD

OJV63954.1 IIEVASKITPNLDLDMALFLENTLTKKGAKLIKGQSINHIEGN----QVVLSSGNKLKAD

WP_066068147.1 IIEKQEKITPNLDEDMALFLENMLLKKNIKIIKGSFISQIQDD----KVVLDNGKEIKSD

WP_069999727.1 IIEKDSKITPNLDEDMAAFLENTLIKKNITILKNNSITTISEK----SVILQDGTEVTSD

SDP09337.1 VVEKQGKITPNLDEDMATFLEKALIKKNISIIKNADIIEILEN----KVLLEDGTEIKSD

WP_023977032.1 IVEMQNKITPNLDEDMAAFLENALIKKKINIIKNSTIIKIDDE----SVTLNDNTVLKSD

SFD29588.1 IVEMQNKITPNLDEDMALFLENTLLKKNINIIKNSTIVKIDDE----SVTLNDNTVLKSD

KAJ53726.1 IIEKQNKITPNLDEDMAAFLENELIKKNMNIIKNSSIIEINQS----NVILEDGRKVKSD

WP_039633362.1 IVEKENKITPSLDEDMAAFLENALKKRNITIIKNSSIVEINEG----KVTLDNGKELKCD

WP_017472957.1 IVEKMPKITPNLDQDMAEYLEDQLKKKAITIKKEATITEITEN----QVLLEDGQAFDAE

SFO84805.1 IVEMKPHITSNLDPDMADYLEDKLAKKGIDVRTSKTVQTIRAD----YVLIDEGETIPAD

SFP14577.1 IVEMKSHITSNLDPDMADYLEDKLIKKGIDVRTDTTVKAIQAN----HVKIDETETIPAE

WP_062323720.1 IVEMKPHITSNLDPDMADYLEDKLVKKGIDVRTNKTVKGIQAD----HVKIDEEETIPAE

WP_073271217.1 VVEMLDQVTPGLDEDMAGYVEDHLSENGVEVHTGTSVKEITKE----GVILSSGKKIASQ

WP_031573915.1 MVELLPQVSPGLDEDMAILVEEHLITKGVRVITGKSAKEITEN----EVILSDDDKLPGD

WP_074913255.1 MVELLTQVSPGLDEDMAILVEEHLAAKGVQVITGKSAKEITEN----EVILSDESKLPGD

OGO93293.1 LIERLPQITPGLDSDMSVYVEEHLQKNDVTVFTSTTAIEITDK----TIVLADGTQIDTE

OJU15484.1 LIERLPQVMPGLDSDMAVHVKSEIQKNGVSVFLNAIVTEITES----GIILAGGEQINAD

SCP98331.1 LVERLPQVTPGLDSDMAVYVEDHLRKNGIPVLTGASVTRIEGN----RVILEDEREIEAD

WP_066089511.1 LIERLPQVTPGLDEDMAVYVEEHLQKNQVKLFTGASVVEIKKD----KVILDNGREVAAD

SEW43766.1 LVEKLRQVTPGLDPDMAIYVKDHLEKNGITVLTGVSATEINID----SVVLSDGNMIKAD

WP_014314980.1 MIEKLSQVTPGLDSDMAVYVEEHLKSKGVSVVTGSSIEEITEK----AVILSDSTEVAAD

WP_033166250.1 LIERLPQVTPGLDPDMAVYVEEHLRDKEISVMTDISVKEIFEN----HVILSDDTRLTAD

WP_066648725.1 LLEKLPQVTPGLDSDMAVYVRDHIEKNGVPVLTAVSVVEITES----GVILSDGKEIPAD

WP_020072493.1 LLERLPQVTPGLDADMAIYVQNHIAKNGVSVLTSVSIAEITAK----SVILSDGKEIKAD

WP_014314895.1 LLEKLPQVTPGLDDDMAIYVEEHIRKKGVSVLTSASITEITES----SVLLSDGKEIKAD

WP_023388569.1 MVEKLPQVTPGLDSDMSNYLRDYLEKKGVILHLGTSAAAIQGDGTVSGVTLESGETLQAD

WP_040428848.1 VVEMEDHLMKPLDIDVSVYLKDALLKKGVKIYLDSTVVELQGDMRTNTALLKDGAKIDTD

WP_073016597.1 VVEMEDHLMKPLDKDVSLYLKDTLIKHEVDVILNDGVVKFEGDSVGTKAFLKSGKDIETD

WP_021801810.1 VVEMEDHLMKPLDKDVSLYLKDTLLKHEINVILNDGVVKFEGDSVARRVFLKSGKHIETD

WP_018703748.1 IVEMASQVMPALDADMAIYVEAELRKKGVSVITEDSVVCFEGKDRVKQVVLKSGEKVTVD

WP_034869873.1 LIEKLSQVTPGLDTDMAAYVEKYLKDKGINIILKDSVIELNKGDRTTEVVLQSGKKINGD

WP_073538592.1 LVERLNQVTPGLDSDMAVYVEKYLKDKNINVILDDSVVELNGYGLINQVVLQSGKVLNTD

WP_007062926.1 LVERFNQVTPGLDEDMAVYVEKYLKSKDINMILGDSVVRLKGEEIVNQVVLQSGKTIDTD

WP_066620912.1 LVERLNQVTPGLDSDMAVHVEKYLKSKDINMVLGDSVVELKGEPLVNQVVLQSGKVINSD

WP_004455169.1 LIERLDQVTPGLDSDMAVYVEKYLKSKDINLILGDSVAELKGEPFISEVVLESGKVLDTD

WP_071167487.1 LIERLDQVTSGLDSDMAVYVEKYLKSKDINLILGDSVAELKGEPFISEVVLESGKVLDTD

WP_035776858.1 LVERLNQVTPGIDSDMAVYVEKYLNSKDINLILGDSVTELRGEPFVNEVVLESGKILNTD

WP_015613982.1 LVERLNQVTPGLDSDMAAYVEKYLKSKDINLILGDSVAALKGETLVNEVALESGKVLNTD

WP_026883985.1 VVELAPQVMPPLDKDMATYIERYLIKKTVNLILGDSVASIEGEVENTKLVLSSGNIIEAD

WP_007930621.1 VVEMAGHVMPALDQDMAVYVADHLLEKGVQVIVNDSVIRLEGEPSVSKVILKSGKEIDAD

WP_007952659.1 VVEMAGHVMPALDPDMAVYVADHLVEKGVQVIVNDSVIRLEGEPSVSKVILKSGKEIDAD

::: :: * ::: : : . :

OHD09048.1 FILVAAGVRPNTTLLKEIGIKTGKSGGILVNEKMETSLKDIYAAGDCCEHLSLLTGEMIY

WP_038668288.1 MVILSTGVKPAVDLAKQAGIELGTTGAIKVDTKMETSIKGIYAVGDCSESYFLIDGNPCY

WP_026825315.1 FVILSVGVRPNVELAMQMGVKLGETGAIAVNERMETNVSDVYAAGDVAESFSIVTGKPIW

WP_074036424.1 FVILSVGVRPNVELAMNMGVELGETGAIAVNERMETNIPDVYAAGDVAESFSIVTGKPIW

WP_047794995.1 LVILSVGVRPNVELAMQMGVTLGETGAIAVNERMETNIPDVYAAGDVAESFSIVTGKPIW

WP_074036076.1 LVILSVGVRPNVELAMNMGVKLGETGAIAVNERMETNIPDVYAAGDVAESFSIVTGKPIW

WP_031422658.1 FVILSVGVRPNVELAMNMGVKLGETGAIAVNERMETNIPDVYAAGDVAESFSIVTGKPIW

WP_012727071.1 FVILSVGVRPNVELAMNMGVKLGETGAIAVNERMETNIPDVYAAGDVAESFSIVTGKPIW

WP_016509108.1 LVILSVGVRPNTELAKSIGVTIGETGAIAINERMETNVDGVYAAGDVAESFSLVTGKPIW

WP_070328560.1 LVILSVGVRPNTELAKSIGVTIGETGAIAINERMETNVDSVYAAGDVAESVSLVTGKPIW

WP_071398091.1 LVILSVGVRPNTELAKSIGVTIGETGAIAINERMETNVDGVYAAGDVAESVSLVTGKPIW

WP_034817875.1 LVILSVGVRPNTELAKSIGVEIGETGAIAINERMETNVENVYAAGDVAESFSLVTGKPIW

WP_034804577.1 LVILSVGVRPNTELAKSIGVNIGETGAIAINERMETNVENVYAAGDVAESFSLVTGKPIW

WP_047374565.1 LVILSVGVRPNTELAKSIGVEIGETGAIAINERMETNVDNVYAAGDVAESFSLVTGKPIW

WP_029334490.1 LVILSVGVRPNTELAKSIGVTIGETGAIAINERMETNVDNVYAAGDVAESFSLVTGKPIW

WP_024370588.1 LVILSVGVRPNTELAKSIGVTIGETGAIAINERMETNVENVYAAGDVAESFSLVTGKPIW

WP_021066461.1 LVILSVGVRPNTELAKSIGVTIGETGAIAINERMETNVKNVYAAGDVAESFSLVTGKPIW

WP_029594929.1 LVILSVGVRPNTELAKSIGVTIGETGAIAINERMETNVKNVYAAGDVAESFSLVTGKPIW

WP_058764331.1 LVILSVGVRPNTELAKSIGVTIGETGAIAINERMETNVKNVYAAGDVAESFSLVTGKPIW

WP_034778796.1 LIILSVGVRPNTELAKSIGVTIGETGAIAINERMETNVKNVYAAGDVAESFSLVTGKPIW

WP_039810825.1 LVILSVGIRPNTKLAKGIGVEIGSTGAIKVNKKMQTNVPDVYAVGDVAESFSLITGKPIY

WP_056061304.1 LVILSVGVKPNTQLAQEIGVTIGQTGAIAVNRQMQTNIEGVYAVGDVAESFSLITGQPLY

WP_035408065.1 LVILSVGVKPNTQLAQEIGVTIGQTGAIAVNRQMQTNIKGVYAVGDVAESFSLITGQPLY

WP_029333607.1 FVILSVGVKPNTELATQIGVTLGQTGAIAVNPQMQTNVKDVYAVGDVAESFSVITGEAIY

WP_053453049.1 LVILSVGVKPNTGLAKQIGVALGKTGAIAVNRKMQTNVADVYAVGDVAESFSVITGEAIY

WP_058705130.1 LVILSVGVKPNTDLAKQIGVELGKTGAIAVNRQMQTNVADVYAVGDVAESFSVITGEALY

WP_023467877.1 LVILSVGVKPNTGLAKQIGVELGKTGAIAVNRHMQTNVADVYAVGDVAESFSVITGEALY

WP_058713610.1 LVILSVGVKPNTGLAKQIGVELGKTGAIAVNRYMQTNVADVYAVGDVAESFSVITGEALY

WP_058265269.1 LVILSVGVKPNTGLAKQIGVELGKTGAIAVNRYMQTNVADVYAVGDVAESFSVITGEALY

WP_035397995.1 LVILSVGVKPNTGLAKQIGVELGKTGAIAVNRYMQTNVADVYAVGDVAESFSVITGEALY

WP_064300131.1 LVILSVGVKPNTGLAKQISVVLGKTGAIAVNRHMQTNVADVYAVGDVAESFSVITGEALY

WP_055967341.1 LVILSVGVKPNTGLAKQIGVELGKTGAIAVNRHMQTNVADVYAVGDVAESFSVITGEALY

WP_050677028.1 LVILSVGVKPNTGLAKQIGVELGKTGAIAVNRHMQTNVADVYAVGDVAESFSVITGEALY

WP_029341349.1 LVILSVGVKPNTGLAKQIGVELGKTGAIAVNRHMQTNVADVYAVGDVAESFSVITGEALY

WP_026831288.1 LVILSVGVKPNTELAKQIGVEMGQTGAIAVNRKMQTNLTDVYAVGDVAESFSVITGEAIY

WP_014970324.1 LVILSVGVKPNTDLAKQIGVDIGQTGAIAVNRKMQTNLTDVYAVGDVAESFSVITGEAIY

WP_071500702.1 LVILSVGVKPNTELAKQIGVELGQTGAIAVNRKMQTNLTDVYAVGDVAESFSVITEEAIY

WP_028106360.1 LVILSVGVKPNTELAKQIGVELGQTGAIAVNRKMQTNLTDVYAVGDVAESFSVITGEAIY

WP_026833068.1 LVILSVGVKPNTELAKQIGVELGQTGAIAVNRKMQTKLTDVYAVGDVAESFSVITGEAIY

WP_026828434.1 LVILSVGVKPNTALAKQIGVELGQTGAIAVNRKMQTNLPDVYAVGDVAESFSVITGEAIY

WP_012370312.1 LVILSVGVKPNTELAKQIGVGLGQTGAIAVNRKMQTNVADIYAVGDVAESFSVITGEAIY

WP_071873852.1 VVFLSVGVRPNTKLAKQIGVEIGTTGAIAVNKKMQTNVPDVYAVGDVAESFSVITGKPIY

WP_073414130.1 IVILAAGVRPNTTLAKEAGITLGSTGAIAVNDKMQTNLQDIYAVGDVAESISVITGKPLY

WP_066448669.1 IVLLATGVRPNAQLAKEIGVTIGTSGAIAVNSKMQTNIPNVYAVGDVAESYSVITGKPIY

WP_016201870.1 MVILATGVRPNTKLAKEIGVELGNSGAIAVNSKMQTNLPDIYAVGDVAESYSVITGKSIY

WP_047943166.1 MVILATGVRPNTQLAKEIGVEIGATGAIAVNTKMQTNLPDVYAVGDVAESYSVITGKPIY

WP_016204927.1 IVILATGVRPNTQLAKEIGVEIGSTGAIAVNSKMQTNLPDVYAVGDVAESYSVITGKPIY

WP_053602939.1 LVILAVGVRPNTKLAKEIGAELGNTGAIAVNTKMQTNIPDVYAVGDVAESFSVITEKPIY

WP_010649726.1 MVILAVGVKPNTKLANDIGVKLRTTGAIAVNSKMQTNLTDVYAVGDCAESFSLITGKPIY

WP_074600543.1 MVILATGVRPNSSLAKSIGVELGASGAIKVNTKMQTNVPDVYAVGDVAESFSVVTGNALY

WP_008633299.1 MVILASGVRPNAELAQEIGVELGKSGAVKVNSKMQTNIPDVYAVGDVAESFSILTGEPIY

WP_027953781.1 MVILATGVRPNTKLAEQIGVELGVSGAVQVNEKMQTNLPDVYAVGDVAESYSIVTGNPLY

WP_067725917.1 MVILATGVRPNTKLAQEIGVELGASGAVKVNTKMQTNLPDVYAVGDVAESFSVITGKAIY

WP_071649283.1 MVILATGVRPNTKLARDLGVELGTSGTIKVNTKMQTNLPDIYAVGDVAESFSVITGKPIY

WP_010652163.1 MVILATGVKPNTKLAEKIGIELGASGAIKVNSRMQTNLSDIYAVGDVAESFSIITGKPIY

WP_041844632.1 IVILATGVKPNTKLATEIGIEIGSSGAIKVNNKMQTNLPDVYAVGDVAESFSLITGKPIY

WP_050739125.1 MIIIATGVKPNVSLAEDAGLILGTTGAIKVDEHMMTIDTDVYACGDCIETWSAVTKKPHY

SEO96011.1 LVIMATGIKPNVQLAERAGVELGETGAIKVNKRMQTNMEGIYACGDCIETFSLITMQPVY

WP_069871897.1 LVVMATGVRPNTVLATTIGIELGKTGAIKVDKQMKTNVSDIYACGDCIETYSAITNKPVY

OJV63954.1 MVFMATGVRPNTALATSAGIKLGKTGAIKVDQQMKTNVADIYACGDCVETFSAITNKPVY

WP_066068147.1 MVIMATGVRPNVKLAKEAGVELGVTGAIKVNSKMQTNIADVYACGDCIETFSLITSKPVY

WP_069999727.1 MVIMAAGVRPNVTLAKEAGIVLGATGAIKVDHQMKTSVKDIYACGDCIETFSVITGKPVY

SDP09337.1 MVIMATGVKPNVSLAKEAGVEIGITGAIKVDTSMRTNIEDIYACGDCIETFSSITGKAVY

WP_023977032.1 MVIMATGVRPNTEIAREAGIEIGVTKAIKVNNKMQTNITDIYACGDCIETFSTITGKPVY

SFD29588.1 MVIMATGVRPNTELAREAGIEVGVTKAIKVNNKMETNITDIYACGDCIETFSSITGKPVY

KAJ53726.1 MVIMATGIKPNVTLAKKAGVEIGVTGAIKVSTKMETSIKDIYACGDCIETFSIVTGKPVY

WP_039633362.1 MTIMATGVKPNIKLARDAGIEIGITSAIKVTTKMETNIKDIYACGDCIETFSMVTGKPIY

WP_017472957.1 LVIMATGVKPNVKVAEQMGVKLGSTGAIQVNTTMQTNVPDVYACGDCIETFSMLTGKAVY

SFO84805.1 LVILATGVKPNVTLAKAIGIEIGQTGAIKVNERMETNIKNIYSCGDCIETFQRQTNQPVY

SFP14577.1 LVILATGVKPNVALAKAIGVEIGETGAIKVNERMETNLKNVYSCGDCIETFQRQTNKPVY

WP_062323720.1 LVILATGVKPNVELAKAIGVEIGETGAIKVNERMETNLKNIYSCGDCIETFQRQTHKPVY

WP_073271217.1 MIILATGVRPNTKLAKEAGIHLGDSGGIKVDKKMETNIKDIYACGDCIEHYHVITKKPIY

WP_031573915.1 LVIIATGVRPHVELAKDAGIELGVTGAIKVNPFTETSVADIYAAGDCMEQYHSITGKPVY

WP_074913255.1 LVIVATGVRPHVELAKEAGIEMGVTGAIKVNPFMETSVADIYAAGDCIEQYHSITGKPVY

OGO93293.1 LVIVAVGIRPNTEFAKAAGIELGIAGAIKVNERMQTNIQDIYACGDCIEHKSLITGNPIY

OJU15484.1 LVLISAGVQPNTELAFSAGVRLGITGAIQVNTKMQTNIDNIYACGDCTEQYHLVTGKPVY

SCP98331.1 FVLVSTGVKPNTDLAVRAGIELGITKAIQVNEKMMTNIEDIYACGDCAEQYHVVTGKPVY

WP_066089511.1 FVLLSTGVRPNTDLAVKAGIELGATRAIKVDNRMMTNVKDIYSCGDCSEQFHVVTGKPVY

SEW43766.1 MVLISVGVRPNTAIATQAGIELGITKAIKVDEKMRTNIEDIYACGDCIEHFNIVTGSPTY

WP_014314980.1 VVLLSTGVRPNSELAKSAGIELGVTGAIKVNRRMETSQKDIYACGDCIEQFHVVTGKPVY

WP_033166250.1 MVLLFTGVRPNIELAKAAGIELGETGAIKVNRKMETNRKNIYACGDCIEQFHVVTGKPVY

WP_066648725.1 LVLISAGVRPNTVLAKAAGIELGVSGAIRVNTKMQTSQPDIYACGDCIEQFHTVTGKPVY

WP_020072493.1 IVLVSTGVRPNTELAATAGIGLGVSGAIRVNTRMETSQKDIYACGDCIEQFHVVTGKPVY

WP_014314895.1 MVLVSTGVRPNTELAAAAGIQLGVAGAIRVNTRMQSSIPDIYACGDCIEQLHLITGKPVY

WP_023388569.1 MVILAIGVRPETTLARNCGIETGQSGAIRTGRSMKTSINDIYACGDCAESFSVITGKPVY

WP_040428848.1 LVIVATGIKPNVELAKEAGIELGVTGAIKVNEKMETSIKGIYACGDCAESYSLITGKPIY

WP_073016597.1 MVIVATGVRPNVELAKNCGVEIGETGAIKVNTKKQTNIKDIYACGDCAESYSIVTGKNFY

WP_021801810.1 MVLVATGVRPNVELAKNAGVEIGETGAIKVNNKMQTNIEDIYACGDCAESYSIVTGKHFY

WP_018703748.1 MVIVAAGVQPNVSLAQQAGVKLGVTGAIEVNAKMQTSALDVYACGDCAEGFSLLTQAPFY

WP_034869873.1 FVIMAIGVKPNVELAKEAGIEIGVTGAIKVNKNMQTNYEDIYSCGDCTEGFSLITGKPLF

WP_073538592.1 FVIMSIGVKPNVEIAKEAGIELGAKGAIKVNTKMMTNIENVYACGDCAESYSVITKKSFY

WP_007062926.1 FVVMSIGVKPNTELAKEAGIELGINGSIKVNKKMMTNIKDVYACGDCAESYSIITGKPLY

WP_066620912.1 FVIMSIGVKPNVELAKEAGIELGANGSIKVDTKMMTNIKDVYACGDCAESYSVITKKPLY

WP_004455169.1 FVIMSIGVKPNTELAKNAGIELGAKGSIRVNTKMMTNIKDIYACGDCTESFSVINEKSLY

WP_071167487.1 FVIMSIGVKPNTELAKNAGIELGAKGSIRVNTKMMTNIKDIYACGDCTESFSVINEKSLY

WP_035776858.1 LVIMSIGVRPNVEIAKKAGIELGANGSIRVNTKMMTNIENIYACGDCTESFSVINKKPLY

WP_015613982.1 FVIMSIGVKTNTELARNAGIDLGAKGSIRVNTKMMTNIEDIYACGDCTESFSVINKKPLY

WP_026883985.1 FVIMAVGIKPNAMIAKKAGIELGESGAIKVNIKMQTNYDYIYACGDCAESYSLITGQDIY

WP_007930621.1 FVIMAVGVRPNIELAQKSGIELGPTGAIKVNRKMQTNIDDIYACGDCAESYSLMTGKPLY

WP_007952659.1 FVIMAVGVRPNVDLAQKAGIELGPTGAIKVNRKMQTNLDEIYACGDCAESYSLMTGKPLY

. .: *::. . . : : :*: ** * :

OHD09048.1 RPMGSTANKMGRILGDGLTGGDLRFQGILGTGICRIFDKTIASTGMTEKEALSKGYDIEV

WP_038668288.1 RPMGSTANKTGRIAGDVITGGDLEFRGILGTGIVKVFDLTCGQTGYTEKEARRAGYDVEI

WP_026825315.1 RPLGSTANKMGRTAGDIITGGHLEHRGILGTGIFKVFGLAVAQTGLAEKEARELGYDIEV

WP_074036424.1 RPLGSTANKMGRTAGDVITGGHLEHRGILGTGIFKVFGLAVAQTGLSEKEAREHGYDIEV

WP_047794995.1 RPLGSTANKMGRTAGDVITGGHLEHRGILGTGIFKVFGLAVAQTGLAEKEARELGYDIEV

WP_074036076.1 RPLGSTANKMGRTAGDVITGGHLEHRGILGTGIFKVFGLAVAQTGLAEKEARELGYDIEV

WP_031422658.1 RPLGSTANKMGRTAGDVITGGHLEHRGILGTGIFKVFGLAVAQTGLAEKEARELGYDIEV

WP_012727071.1 RPLGSTANKMGRTAGDVITGGHLEHRGILGTGIFKVFGLAVAQTGLAEKEARELGYDIEV

WP_016509108.1 RPLGSTANKMGRALGDLLTGGELEHRGILGTGIFKVFGLAVAQTGLSEREARDLGYDVEV

WP_070328560.1 RPLGSTANKMGRSLGDLLTGGELEHRGILGTGIFKVFGLAVAQTGLSEREARDLGYDVEV

WP_071398091.1 RPLGSTANKMGRALGDLLTGGELEHRGILGTGIFKVFGLAVAQTGLSEREARELGYDVEV

WP_034817875.1 RPLGSTANKMGRALGDLLTGGDLEHRGILGTGIFKVFGQAVAQTGLSEREARELCYEVEV

WP_034804577.1 RPLGSTANKMGRALGDLLTGGDLEHRGILGTGIFKVFGQAVAQTGLSEREARELGYDVEV

WP_047374565.1 RPLGSTANKMGRALGDLLTGGDLEHRGILGTGIFKVFGLAVAQTGLSEREARELGYDVEV

WP_029334490.1 RPLGSTANKMGRALGDLLTGGDLEHRGILGTGIFKVFGLAVAQTGLSEREARELGYDVEV

WP_024370588.1 RPLGSTANKMGRALGDLLTGGDLEHRGILGTGIFKVFGLAVAQTGLSEREARELGYDVEV

WP_021066461.1 RPLGSTANKMGRALGDLITGGDLEHRGILGTGIFKVFGQAVAQTGLSEREARELGYDVEV

WP_029594929.1 RPLGSTANKMGRALGDLITGGDLEHRGILGTGIFKVFGQAVAQTGLSEREARELGYDVEV

WP_058764331.1 RPLGSTANKMGRALGDLITGGDLEHRGILGTGIFKVFGQAVAQTGLSEREARELGYDIEV

WP_034778796.1 RPLGSTANKMGRALGDLITGGDLEHRGILGTGIFKVFGQAVAQTGLSEREARELGYDIEV

WP_039810825.1 RPLGSTANKTGRIAGQVITGEEAEHRGILGSGIFKVFDLTVAQTGLTEREAVEEGYEIEV

WP_056061304.1 RPLGSTANKMGRIAGMVITGEAIEHRGILGTGIFKAFDLTVAQTGLTEKEARDAGYDIEV

WP_035408065.1 RPLGSTANKMGRIAGMVITGEAIEHRGILGTGIFKAFDLTVAQTGLTEKEARDAEYDIEV

WP_029333607.1 RPLGSTANKMGRIAGMVITGEEAAHRGILGTGIFKAFDLTVAQTGLTEKEALAAGYDIEI

WP_053453049.1 RPLGSTANKMGRIAGMVITGEEAEHRGILGTGIFKAFDLTVAQTGLTEKEARESGYDIEV

WP_058705130.1 RPLGSTANKMGRIAGMVITGEEAEHRGILGTGIFKAFDLTVAQTGLTEKEAHASGYDIEV

WP_023467877.1 RPLGSTANKMGRIAGMVITGEEAEHRGILGTGIFKAFDLTVAQTGLTEKEARESGYDIEV

WP_058713610.1 RPLGSTANKMGRIAGMVITGEEAEHRGILGTGIFKAFDLTVAQTGLTEKEARESGYDIEV

WP_058265269.1 RPLGSTANKMGRIAGMVITGEEAEHRGILGTGIFKAFDLTVAQTGLTEKEARESGYDIEV

WP_035397995.1 RPLGSTANKMGRIAGMVITGEEAEHRGILGTGIFKAFDLTVAQTGLTEKEARESGYDIEV

WP_064300131.1 RPLGSTANKMGRIAGMVITGEEAEHRGILGTGIFKAFDLTVAQTGLTEKEARESGYDIEV

WP_055967341.1 RPLGSTANKMGRIAGMVITGEEAEHRGILGTGIFKAFDLTVAQTGLTEKEARESGYDIEV

WP_050677028.1 RPLGSTANKMGRIAGMVITGEEAEHRGILGTGIFKAFDLTVAQTGLTEKEARESGYDIEV

WP_029341349.1 RPLGSTANKMGRIAGMVITGEEAEHRGILGTGIFKAFDLTVAQTGLTEKEARESGYDIEV

WP_026831288.1 RPLGSTANKMGRIAGMVITGEEAEHRGILGTGIFKAFDLTVAQTGLTEKEAREAGYDIEV

WP_014970324.1 RPLGSTANKMGRIAGMVITGEEAEHRGVLGTGIFKAFDLTVAQTGLTEKEAREAGYDIEV

WP_071500702.1 RPLGSTANKMGRIAGMVITGEEAEHRGILGTGIFKAFDLTVAQTGLTEKEAREAGYDIEV

WP_028106360.1 RPLGSTANKMGRIAGMVITGEEAEHRGILGTGIFKAFDLTVAQTGLTEKEAREAGYDIEV

WP_026833068.1 RPLGSTANKMGRIAGMVITGEEAEHRGILGTGIFKAFDLTVAQTGLTEKEAREAGYDIEV

WP_026828434.1 RPLGSTANKMGRIAGMVITGEEAEHRGILGTGIFKAFDLTVAQTGLTEKEAREAGYDIEI

WP_012370312.1 RPLGSTANKMGRIAGMVITGEEAEHRGILGTGIFKAFDLTVAQTGLTEKEAREAGYDIEV

WP_071873852.1 RPLGSTANKMGRIAGDVITGGNLEHRGVLGTGIFRIFDLHVGQTGLTEKEAIQEGYEYET

WP_073414130.1 RPLGSTANKMGRIAGDVITGGDLAHRGILGTGILRVFNLAVGQTGITEKEAKEHGYHVDI

WP_066448669.1 RPLGSTANKMGRIAGDVITGGSLEHRGILGTGILRVFDLAVGQTGMNEVEALEEGYDIEI

WP_016201870.1 RPLGSTANKMGRIAGDVITGGSLEHRGILGTGILRVFDLAVGQTGMNEVEALEEGYDIEI

WP_047943166.1 RPLGSTANKMGRIAGDVITGGSLEHRGILGTGILRVFDLAVGQTGMNEVEALEEGYNIEI

WP_016204927.1 RPLGSTANKMGRIAGDVITGGSLEHRGILGTGILRVFDLAVGQTGMNEVEALEEGYDIEI

WP_053602939.1 RPLGSTANKMGRIAGDVITGGNLEHRGILGTGILRVFDFAVGQTGLNEKEARKEGYDIEV

WP_010649726.1 RPLGSTANKMGRIAGDVITGGTLDHRGILGTGILRVFDLAVGHTGLSEKEALQLGYDVEV

WP_074600543.1 RPLGSTANKMGRIAGDVITGGDLEHRGILGTGILRVFDLAVGYTGLSERDALAEGFDIEV

WP_008633299.1 RPLGSTANKMGRIAGDVITGGDLEHRGILGTGILRVFDLAVGYTGFSEKEAKEKGYDLEV

WP_027953781.1 RPLGSTANKMGRIAGDVITGGDLAHRGILGTGILRVFDLAVGYTGLSEKDAVMEGYDIEV

WP_067725917.1 RPLGSTANKMGRIAGDVITGGDLEHRGILGTGILRVFDLAVGYTGLSEKEALQEGFDIEV

WP_071649283.1 RPLGSTANKMGRIAGDVITGGDLEHRGILGTGILRVFDLAVGYTGLSEKEAKAEGFDLEV

WP_010652163.1 RPLGSTANKMGRIAGDVITGGDLEHRGILGTGILRVFNLAVGYTGLSEKEALDEGFDTEV

WP_041844632.1 RPLGSTANKMGRIAGDVITGGNLEHKGILGTGILRVFDLTVGYTGLSEKEALAEGFDIEV

WP_050739125.1 RPLGSTANKTGRICGDSITGGNMIYTGNLGTGIFKFFDLSIGSTGLSEAEAIKNGYDFLV

SEO96011.1 HPLGSTANKTGRIAGDVLSGGSLFYKGNLATGIYRLFNLTVATTGLSENEAKESGINVVA

WP_069871897.1 RPLGSTANKTGRIAGDALTGGLLEYQGNLSTGIFKLFDLTIASTGLSEKEARDEGYDIEI

OJV63954.1 RPLGSTANKTGRIAGDALTGGKLRYQGNLSTGIFKLFDLTIATTGLSEKEAIDEGYDIEI

WP_066068147.1 RPLGSTANKTGRIAGDVLTGGNLEYRGNLGTGIFKLFDIAVSNTGLTEKEAAELGYDVVV

WP_069999727.1 RPLGSTANKTGRIAGDALTGGSLSYRGNLSTGIFKLFDLTVANTGLTEKEALQEGYDIVI

SDP09337.1 RPLGSTANKTGRIAGDVVTGGKLRYRGNLSTGIFKLFDMAIGSTGLSEKEALEEGYEIVM

WP_023977032.1 RPLGSTANKTGRIAGDNITGGNIEYRGNLSTGIFKLFDMTIANTGLSEKEALEEGYDIQI

SFD29588.1 KPLGSTANKTGRIAGDNATNGNLEYKGNLGTGIFKLFDLTIANTGLSEKEAIQEGYEIQI

KAJ53726.1 RPLGSTANKTGRIAGDVVTGGNLEYKGNLGTGIFKLFNMTIANTGLTEMEAIKEGYEIAI

WP_039633362.1 RPLGSTANKTGRIAGDVVTDGTLEYRGNLSTGIFKIFNITVANTGLSEKEAICEGYDIAI

WP_017472957.1 RPLGSTANKTGRIAGDVLSGGTLEYRGNLGTSIYKLFDLTIANTGLTEQEAEDAGYNVVV

SFO84805.1 RPLGSTANKTGRIAGDQLSGGSLEFKGILGTSIYKLFEFAIANTGLSEIEARDAGYDVEI

SFP14577.1 RPLGSTANKTGRIAGDQLSGGSLEFKGILGTSIYKLFEFTIANTGLSEVEARAEGFDIEV

WP_062323720.1 RPLGSTANKTGRIAGDQLSGGSLEFKGILGTSIYKLFEFTVANTGLSEAEARAEGFNIEI

WP_073271217.1 RPLGSTANKTGRIAGENITGGNLEFRGILGTGIFRVFDLAVGQTGLSEKEAKSMGYDVEI

WP_031573915.1 RPLGSTANKTGRIAGNNATGGNLEFRGVLGTGIYKIFDLAVAQTGLTEREALKEGYEISV

WP_074913255.1 RPLGSTANKTGRMAGNNATGGNLEFRGVLGTGIYKIFDLAVAQTGLTEREALKEGYEISV

OGO93293.1 RPLGSTANKTGRIAGDCVTGGSLSFRGILGTGIFRIFDMTVAQTGLSEREAIELGYDFIV

OJU15484.1 RPLGSTANKTGRIAGDSITGGSLEFRGILGTGIFQIFDLTVAQTGLTEREAVEQGFNVQV

SCP98331.1 RPMGSTANKTGRIAGDVVTGGDLAFRGILGTGIFQIFGMTVALTGLSEREAQKEGYEVEV

WP_066089511.1 RPMGSTANKTGRIAGDVATGGDLSFRGILGTGIFKVFDMTVALTGLSEREATKEGYNVVF

SEW43766.1 RPLGSTANKTGRIAGDSVTGGDLAFRGILGTGIFQLFGMTIAQTGLSEREATELGYDIAV

WP_014314980.1 RPLGSTANKTGRIAGDSMTGGNLEFRGILGTGIFKIFDMTVAQTGLSEREAHELGYGVEV

WP_033166250.1 RPLGSTANKTGRIAGDSMTGGNLEFQGVLGTGIFKIFDLTVAQTGLSEREAHELGYDVVV

WP_066648725.1 RPLGSTANKTGRIAGDSLTGGSLEFRGILGTGIFRIFGMTVAQTGLSEREALALGYQTAV

WP_020072493.1 RPLGSTANKTGRIAGDSVTGGRLEFRGVLGTGIFKIFDLTVAQTGLSEREATEQGYEVVV

WP_014314895.1 RPLGSTANKTGRIAGDSMTGSDLEFRGILGTGIFKIFDMTVAQTGLSEREAKEQGYDIAV

WP_023388569.1 RPLGSTANKTGRIAGNSIFNDSLEFRGVLGTGIFRVFDMTVGQTGLTEREALQEGCDIEV

WP_040428848.1 RPLGSTANKMGRIAGDSLTGYDLNFRGILGTGIFKIFDYTIAQTGLTEREALEEGFNVSV

WP_073016597.1 RPLGSTANKMGRITGDQITGGDLEFRGILGTGIFKIFDMAVAQTGLTEIEAINEGYDISV

WP_021801810.1 RPLGSTSNKMGRIAGDQITGGDLEFRGIVGTGIFKIFDMAVAQTGLTEVEAINEGYDISV

WP_018703748.1 RPMGSTANKMGRIAGDQATGGMLEFRGVLGTGIVKIFDLTVAHTGMSEQQARKEGYAVVV

WP_034869873.1 RPLGSTANKMGRIAGDSLTGGKLQFRGVLGTAILKVFDMAVAQTGLSEKEAISESFDVVV

WP_073538592.1 RPLGSTANKMGRIAGDQLSGGNLEFRGILGTGIFKIFDMTVAQTGLTEREALNEGFEPVV

WP_007062926.1 RPLGSTANKMGRIAGDQLSGGNLEFRGILGTGIFKIFDMTVAQTGITEREAISEGYEPVI

WP_066620912.1 RPLGSTANKMGRIAGDQVSGGNLEFRGILGTGIFKLFDMTVAQTGLTEREALNEGFETVI

WP_004455169.1 RPLGSTANKMGRISGDQLTGGSLEFRGILGTGIFKVFDMTVAQTGFTEKEAQSEGFDTVV

WP_071167487.1 RPLGSTANKMGRISGDQLTGGSLEFRGILGTGIFKVFDMTVAQTGFTEKEAQSEGFDTVV

WP_035776858.1 RPLGSTANKMGRIAGDQLNGGSLEFRGILGTGIFKVFDMTVAQTGLTEKEAQSEGFDTVV

WP_015613982.1 RPLGSTANKMGRIAGDQLTGGNLEFRGILGTGIFKVFDMAVAQTGLTEKEAQSEGFDTVV

WP_026883985.1 RPLGSTANKTGRIVGDSITGGALEFRGILGTGIFKVFDLAVAYTGLSEKEAIKEGYEVVV

WP_007930621.1 RPLGSTANKMGRITGDQITGGDLEFRGILGTGIFKMFDMAVGQTGLTEKEAKKEGYEVVV

WP_007952659.1 RPLGSTANKMGRITGDQITGGDLEFRGILGTGIFKVFDMAVGQTGLTEKEAKKEGYEVVV

:*:***:** ** * . . * :.:.* : * . ** * :*

OHD09048.1 IHNIKENQSSYLPESSEMLIKAVADKKTETLLGVQIFGGKGVDKRIDVFAAAISFKAKTK

WP_038668288.1 IHNIKPNQTEYFEGSSEMIIKAVADRATQKLLGVQIIGKKGVDKRIDIFATAITFGAKAG

WP_026825315.1 LHNTKPDKPAYMGG-KDMTIKAVADRATRQLLGVQIVGFEGVDKRIDVFVTAITLKAKVD

WP_074036424.1 LHNTKPDKPAYMGG-KDMTIKAVADRSTRQLLGIQIVGFEGVDKRIDVFVTAITLKAKVD

WP_047794995.1 LHNTKPDKPAYMGG-KDMTIKAVADRATRQLLGVQIVGFEGVDKRIDVFVTAITLKAKVD

WP_074036076.1 LHNTKPDKPAYMGG-KDMTIKAVADRATRQLLGVQIVGFEGVDKRIDVFVTAITLKAKVD

WP_031422658.1 LHNTKPDKPAYMGG-KDMTIKAVADRATRQLLGVQIVGFEGVDKRIDVFVTAITLKAKVD

WP_012727071.1 LHNTKPDKPAYMGG-KDMTIKAVADRATRQLLGVQIVGFEGVDKRIDVFVTAITLKAKVD

WP_016509108.1 LHNTKPDKPAYMGG-KDMTIKAIGDRATRQLLGVQIVGFEGVDKRIDVFVTAITFKAKVD

WP_070328560.1 LHNTKPDKPAYMGG-KDMTIKAVGDRATRQLLGVQIVGFEGVDKRIDVFVTAITFKAKVD

WP_071398091.1 LHNTKPDKPAYMGG-KDMTIKAIGDRATRQLLGVQIVGFEGVDKRIDVFVTAITFKAKVD

WP_034817875.1 LHNTKPDKPAYMGG-KDLTIKAIGDRATRQLLGVQIVGFDGVDKRIDVFVTAMTFKAKVD

WP_034804577.1 LHNTKPDKPAYMGG-KDLTIKAIGDRATRQLLGVQIVGFEGVDKRIDVFVTAMTFKAKVD

WP_047374565.1 LHNTKPDKPAYMGG-KDLTIKAIGDQATRQLLGVQIVGFEGVDKRIDVFVTAMTFKAKVD

WP_029334490.1 LHNTKPDKPAYMGG-KDLTIKAIGDRATRQLLGVQIVGFEGVDKRIDVFVTAMTFKAKVD

WP_024370588.1 LHNTKPDKPAYMGG-KDLTIKAIGDQATRQLLGVQIVGFEGVDKRIDVFVTAMTFKAKVD

WP_021066461.1 LHNTKPDKPAYMGG-KDMTIKAIGDRVTRQLLGVQIVGFEGVDKRIDVFVTAITFKAKVD

WP_029594929.1 LHNTKPDKPAYMGG-KDLTIKAIGDRSTRQLLGVQIVGFEGVDKRIDVFVTAITFKAKVD

WP_058764331.1 LHNTKPDKPAYMGG-KDMTIKAIGDRATRQLLGVQIVGFEGVDKRIDVFVTAITFKAKVD

WP_034778796.1 LHNTKPDKPAYMGG-KDMTIKAIGDRATRQLLGVQIVGFEGVDKRIDVFVTAITFKAKVD

WP_039810825.1 LHNIKPDRPEYLGG-KEMVIKAIADRNTGKLLGAQIVGPQGVDKRIDVFVTAISFGAKAE

WP_056061304.1 LHNIKPDRPEYLGG-KEMVIKAIADRNDGRVLGAQIVGPQGVDKRIDVLATAISFKAKAE

WP_035408065.1 LHNIKPDRPEYLGG-KEMVIKAIADRNDGRILGAQIVGPQGVDKRIDVLATAISFKAKAE

WP_029333607.1 LHNIKPDRPEYMGG-KDMVIKAVADRETGRVLGAQIVGPQGVDKRIDVLATAITFKAKAE

WP_053453049.1 LHNIKPDRPEYMGG-KEMTIKAIADRATGRVLGAQIVGPQGVDKRIDVLATAITFKAKAE

WP_058705130.1 LHNIKPDRPEYMGG-KEMTIKAIADRATGRVLGAQIVGPQGVDKRIDVLATAITFKAKAE

WP_023467877.1 LHNIKPDRPEYMGG-KEMTIKAIADRATGRVLGAQIVGPQGVDKRIDVLATAITFKAKAE

WP_058713610.1 LHNIKPDRPEYMGG-KEMTIKAIADRATGRVLGAQIVGPQGVDKRIDVLATAITFKAKAE

WP_058265269.1 LHNIKPDRPEYMGG-KEMTIKAIADRATGRVLGAQIVGPQGVDKRIDVLATAITFKAKAE

WP_035397995.1 LHNIKPDRPEYMGG-KEMTIKAIADRAKGRVLGAQIVGPQGVDKRIDVLATAITFKAKAE

WP_064300131.1 LHNIKPDRPEYMGG-KEMTIKAIADRATGRVLGAQIVGPQGVDKRIDVLATAITFKAKAE

WP_055967341.1 LHNIKPDRPEYMGG-KEMIIKAIADRATGRVLGAQIVGPQGVDKRIDVLATAITFKAKAE

WP_050677028.1 LHNIKPDRPEYMGG-KEMTIKAIADRATGRVLGAQIVGPQGVDKRIDVLATAITFKAKAE

WP_029341349.1 LHNIKPDRPEYMGG-KEMTIKAIADRATGRVLGAQIVGPQGVDKRIDVLATAITFKAKAE

WP_026831288.1 LHNIKPDRPEYMGG-KEMVIKAIADRATGRVLGAQIVGPQGVDKRIDVLATAITFKAKAE

WP_014970324.1 LHNIKPDRPEYMGG-KEMVIKAIADRATGRVLGAQIVGPQGVDKRIDVLATAITFKAKAE

WP_071500702.1 LHNIKPDRPEYMGG-KEMTIKAIADRATGRVLGAQIVGPQGVDKRIDVLATAITFKAKAE

WP_028106360.1 LHNIKPDRPEYMGG-KEMVIKAIADRATGRVLGAQIVGPQGVDKRIDVLATAITFKAKAE

WP_026833068.1 LHNIKPDRPEYMGG-KEMVIKAIADRAIGRVLGAQIVGPQGVDKRIDVLATAITFKAKAE

WP_026828434.1 LHNIKPDRPEYMGG-KEMTIKAIADRATGRVLGAQIVGPQGVDKRIDVLATAITFKAKAE

WP_012370312.1 LHNIKPDRPEYMGG-KEMTIKAIADRATGRVLGAQIVGPQGVDKRIDVLATAITFKAKAE

WP_071873852.1 LYNLKPDHAEYLGG-QELTIKALADRKTGLVLGAQVIGKGGVDKRVDVLATAITFGAKAE

WP_073414130.1 LHNIKPAKAEYLGG-KELVIKAVADRKSGRILGVQIVGEEGVDKRIDVFATAITFKAKAE

WP_066448669.1 LHNIKPSRAEYLGG-KELVIKAIADRDTGRVLGVQIIGEDGVDKRIDVFVTAITFKAKAE

WP_016201870.1 LHNIKPARAEYLGG-KELLIKAIADRQTGRILGVQIVGKEGVDKRIDVFVTAITFKAKAE

WP_047943166.1 LHNIKPARAEYLGG-KELVIKAIADRETGRILGVQIVGEEGVDKRIDVFVTAITFKAKAE

WP_016204927.1 LHNIKPARAEYLGG-KELVIKAIADRETGRILGVQIVGEEGVDKRIDVFVTAMTFKAKAE

WP_053602939.1 LHNLKPAKAEYLGG-KELVIKAVADRASGRILGVQAVGEEGVDKRIDVFATAITFKAKAE

WP_010649726.1 LHNIKPASADYLGG-RELVIKALADRETGRVLGVQIVGVNGVDKRIDVFATAITFNAKAE

WP_074600543.1 LHNIKPARAEYLGG-KELVIKAIADRKTSRILGVQIVGEEGVDKRIDVFVTAISFKAKAE

WP_008633299.1 LHNIKPSRADYLGG-KAIVIKAVADRKTGRILGVQAIGEDGVDKRIDVFVTAISFGAKAQ

WP_027953781.1 LHNIKPARAEYLGG-KEMVIKAIADRKTGRILGVQIVGEDGVDKRIDVFVTAISLKASAE

WP_067725917.1 LHNIKPSRADYLGG-KEMVIKAIADRKTSRVLGVQILGADGIDKRLDVFVTAISFGAKAE

WP_071649283.1 LHNIKPARAEYLGG-KELVIKAIADRKTSQILGVQIVGEDGVDKRIDVFVTAISFKAKAK

WP_010652163.1 LHNIKPARAEYLGG-KELVIKAIADRKTSRILGVQIVGEDGVDKRIDVFVTAISFKAKAE

WP_041844632.1 LHNIKPARADYLGG-KELVIKAIADRKTSQILGVQIIGEEGVDKRIDVFVTAISFKARAG

WP_050739125.1 SHNIKPNKPAYTGG-REMIIKAIADKDTGKILGVQIIGYEGVDKRLDVFVTLITYGATVD

SEO96011.1 AHNSKPDKASYFNG-KEMCIKSVADKQSGRLLGVQIIGEAGVDKRIDVFATLITYGASAD

WP_069871897.1 SHNIKPDKPSYFHG-EEMVIKAIADRASQRLLGVQIIGTTGVDKRIDVFATLITYKAKVE

OJV63954.1 SHNIKPDKPSYFHG-EEMVIKSIADRESQRLLGVQIIGTTGVDKRIDVFATLITYKAKVD

WP_066068147.1 CHNIKPDKPTYFNG-KEMIIKAVADKNTKKILGVQIIGFDGVDKRTDVFATLITYGAKVD

WP_069999727.1 CHNIKPDKPSYFQG-KEMVIKAIADKKTQKLLGVQIIGYEGVDKRIDVFATLITYGAKVD

SDP09337.1 CHNIKPDKPSYFKG-KEMVIKAIADKKTEKILGVQIVGYEGVDKRLDVFVTLITYGAKVD

WP_023977032.1 CHNIKPDKPSYFHG-KEMVIKAIADKKTEKILGVQIVGYEGVDKRIDVFATLITYGAKVD

SFD29588.1 CHNIKPDKPAYFNG-KEMVIKAIADKNTEKILGVQIVGYEGVDKRIDVFATLITYGAKVD

KAJ53726.1 CHNIKPDKPSYFQG-KEMVIKAVADRKTEKLLGVQIVGYDGVDKRIDIFATLITYGAKVN

WP_039633362.1 CHNIKPDKPTYFNG-REMIIKAIADKKTERILGVQIIGYEGVDKRIDVFATLITYGAKID

WP_017472957.1 SHNIKPDKPAYFNG-QEMCIKSIADRETGRLLGAQIVGREGVDKRIDVLVTLITYGAKAE

SFO84805.1 SHNIKPDKPAYFNG-TEMNIKAIADRESKKLLGVQIVGQAGVDKRIDVFSTLITYGATVD

SFP14577.1 SHNIKPDKPAYFNG-TEMNIKAIADRASKKLLGVQIVGQEGVDKRIDVFSTLITYGATVD

WP_062323720.1 SHNIKPDKPAYFNG-TEMNIKAIADRDTKKLLGVQIVGQEGVDKRIDVFSTLVTYGATVD

WP_073271217.1 SHNIKPSRPEYMGG-KEIVIKAIADRKTGRVLGVQVIGEDGVDKRVDVFATAITFGAKAE

WP_031573915.1 SHNIKPNRPEYMGG-KEMVIKSVADKKDGRLLGVQIIGPEGVDKRVDVFAALITFGAKVQ

WP_074913255.1 SHNIKPNRPEYMGG-KEMVIKSVADKKDGRLLGVQIIGPEGVDKRVDVFAALITFGAKVQ

OGO93293.1 SYNIKPDKPEYMGG-KEMVIKTVADKNTGRILGVQIVGFGGVDKRIDVFVTAITFGAKAE

OJU15484.1 CHNIKPDRPEYMGG-KEMVIKGIADKNSGRLLGVQIVGHGGVDKRMDVFVTAITFGAKVE

SCP98331.1 CHNIKPNKPEYMGG-QEMVIKGIADRATGRMLGVQIVGFEGVDKRIDVFVTAITFKAKAE

WP_066089511.1 CHNTKPNKPEYMGG-KEMVIKGIADKDTGRLLGVQIVGFEGVDKRVDVFATAISYKAKVE

SEW43766.1 CHNIKPNKPEYMGG-KEMVIKAVADKSNGRLVGVQIVGFEGVDKRIDVFVTAITFKAKVE

WP_014314980.1 CHNIKPNKPEYMGG-KEMVIKGIADNVTGRLLGAQIIGFEGVDKRIDVFVTAITFKAMVE

WP_033166250.1 CHNIKPNKPEYMGG-KEMVIKGIADKATGRLLGVQIIGYEGVDKRIDVFVTAISFKAKVE

WP_066648725.1 CHNIKPNRPEYMGG-REMVIKGIADRVSGRLLGAQIVGYEGVDKRIDVFVTAITFKAKVE

WP_020072493.1 CHNIKPDKPEYMGG-KEMVIKGIADKNSGRLLGVQIIGSTGVDKRIDIFATAITFKAQVK

WP_014314895.1 CHNIKPNKPEYMGG-KEMVIKGIADKVSGRLLGAQIIGFEGVDKRIDVLVTAITFNAKVE

WP_023388569.1 SHNIKPDRPTYYGG-KEMVIKSIADRKTGKLLGVQIVGYGGVDKRIDVFATAISFGARAE

WP_040428848.1 CHNIKPDKPEYFHG-EEMVIKAVADRHNGRLLGVQIFGKSGVDKRIDVFATALTFGAKVQ

WP_073016597.1 SHNIKPDKPPYYHG-EEMLIKAVADKKTEKLLGVQIVGKAGVDKRIDVFVTAITFGAKVS

WP_021801810.1 SHNIKPDKPPYYHG-EEMLIKAVADKKTEKLLGVQIVGKAGVDKRIDVFVTAITFGAKVS

WP_018703748.1 CHNIKPDKPEYFHG-QEMVIKGIADETTGRLLGVQIVGKSGVDKRMDVFVTAISFGAHVE

WP_034869873.1 CHNIKLDKPTYYNG-KEMIIKGIADKKTGKLLGAQIVGYGGVDKRIDVFVTAITYGANVE

WP_073538592.1 CHNIKPDKPTYYNG-KEMVIKAVADKKTRKLLGVQIVGYAGVDKRVDVFVTAITFGAKVE

WP_007062926.1 CHNIKTDKPEYYHG-KEMIIKAIADKNTGKLLGVQIVGYTGVDKRIDVFITAITFGAKVE

WP_066620912.1 CHNIKPDKPEYYHG-KEMVIKAVADKNTGKLLGVQIVGYTGVDKRVDVFVTAITFGAKVE

WP_004455169.1 CHNIKPDKPAYYHG-KEMVIKAVADKNTGKILGAQIVGYTGVDKRIDVFVTAITFGAKAE

WP_071167487.1 CHNIKPDKPAYYHG-KEMVIKAVADKNTGKILGAQIVGYTGVDKRIDVFVTAITFGAKAE

WP_035776858.1 CHNIKPDKPKYYHG-KEMVIKAVADRNTGKLLGVQIVGYTGVDKRIDVFVTAITFGAKVE

WP_015613982.1 CHNIKPDKPAYYHG-KEMVIKAVADKNTGKLLGVQIVGYTGVDKRIDVFVTAITFGAKAE

WP_026883985.1 CHNIKPDKAIYYGG-EELTIKAVADKNTSKLLGVQIVGTNGVDKRIDVFATAITFGAKVE

WP_007930621.1 CHNIKPDKPEYYHG-SEMVIKAVADKNTGKVLGVQIVGRSGVDKRIDVFVTAITFGAKAE

WP_007952659.1 CHNIKPDKPEYYHG-SEMVIKAVADKNTGKVLGVQIVGKSGVDKRMDVFVTAITFGAKAE

:* * . * : ** :.*. ::* * .* *:*** *:: : :: *

OHD09048.1 NLFHLDLAYSPQFATTKDPIHYTGMILTNSFQRERKIITPRELKEN---RKN-WTVIDVR

WP_038668288.1 DLSHLDLAYAPPYSTTKDPVMYTGMILDNALNRGRKIITAQEL---IADRMK-YTVIDVR

WP_026825315.1 DLFHLDLAYAPPFSTTKDPVMYTGMVLSNSIDKAARLITPAELQTRIDAGED-MQIIDTR

WP_074036424.1 DLFHLDLAYAPPFSTTKDPVMYTGMVLSNSIDKAARLITPAELQTRIDAGED-MQIIDTR

WP_047794995.1 DLFHLDLAYAPPFSTTKDPVMYTGMVLSNSIDKAARLITPAELQTRIDAGED-MQIIDTR

WP_074036076.1 DLFHLDLAYAPPFSTTKDPVMYTGMVLSNSIDKAARLITPAELQARIDAGED-MQIIDTR

WP_031422658.1 DLFHLDLAYAPPFSTTKDPVMYTGMVLANSIDKAARLITPAELQARIDAGED-MQIIDTR

WP_012727071.1 DLFHLDLAYAPPFSTTKDPVMYTGMVLANSIDKAARLITPAELQARIDAGED-MQIIDTR

WP_016509108.1 DLFHLDLAYAPPFSTTKDPVMYTGMALANSMDKSARLITPAELQQRIDADEH-MQVIDTR

WP_070328560.1 DLFHLDLAYAPPFSTTKDPVMYTGMALANSMNKSARLITPAELQQRIDADED-IQVIDTR

WP_071398091.1 DLFHLDLAYAPPFSTTKDPVMYTGMALANSMNKSARLITPAELQQRIDADED-IQVIDTR

WP_034817875.1 DLFHLDLAYAPPFSTTKDPVMYTGMALANSMDETARLITPGELQKRIDAGED-MQIIDTR

WP_034804577.1 DLFHLDLAYAPPFSTTKDPVMYTGMALANSMNKSARLITPAELQKRIDAGED-MQIIDTR

WP_047374565.1 DLFHLDLAYAPPFSTTKDPVMYTGMVLANSMDKAARLITPAELQKRIDAGED-MQVIDTR

WP_029334490.1 DLFHLDLAYAPPFSTTKDPVMYTGMVLANSMDEAARLITPAELQKRIDAGED-MQVIDTR

WP_024370588.1 DLFHLDLAYAPPFSTTKDPVMYTGMVLANSMDKAARLITPAELQKRIDAGED-MQVIDTR

WP_021066461.1 DLFHLDLAYAPPFSTTKDPVMYTGMALANSMNQSARLITPAELQQRIDAGED-MQIIDTR

WP_029594929.1 DLFHLDLAYAPPFSTTKDPVMYTGMALANSMNQSARLITPGELQKRIDAGED-MQIIDTR

WP_058764331.1 DLFHLDLAYAPPFSTTKDPVMYTGMVLANSMDKAARLITPAELQKRIDAGED-MQIIDTR

WP_034778796.1 DLFHLDLAYAPPFSTTKDPVMYTGMVLANSMDQSARLITPGELQKRIDAGED-MQIIDTR

WP_039810825.1 DLFHLDLAYAPPFATTKDPVMYTGMALHNSISKKAPLITPQQLVREIENGEA-FQIIDTR

WP_056061304.1 DLFHLDLAYAPPFSTTKDPILYTGMALDNAIKRTARLKTPNALMEEVAKGQV-FQIIDTR

WP_035408065.1 DLFHLDLAYAPPFSTTKDPILYTGMALDNAIKRTARLKTPNALMEEVAKGQV-FQIIDTR

WP_029333607.1 DLFHLDLAYAPPFSTTKDPVLYTGMALDNAIKKTARLVTPETLVEQTATEES-IQIIDTR

WP_053453049.1 DLFHLDLAYAPPFATTKDPVLYTGMALDNAIKKTARLMTPNELIDQVAGGKA-FQIIDTR

WP_058705130.1 DLFHLDLAYAPPFATTKDPVLYTGMALDNAIKKTARLITPNELIDQVASGKS-FQIIDTR

WP_023467877.1 DLFHLDLAYAPPFATTKDPVLYTGMALDNAIKKTARLMTPNELIDQVAGGKS-FQIIDTR

WP_058713610.1 DLFHLDLAYAPPFATTKDPVLYTGMALDNAIKKTARLMTPNELIDQVAGGKS-FQIIDTR

WP_058265269.1 DLFHLDLAYAPPFATTKDPVLYTGMALDNAIKKTARLMTPNELIDQVAGGKS-FQIIDTR

WP_035397995.1 DLFHLDLAYAPPFATTKDPVLYTGMALDNAIKKTARLMTPNELIDQVAGGKS-FQIIDTR

WP_064300131.1 DLFHLDLAYAPPFATTKDPVLYTGMALDNAIKKTARLMTPNELIDQVASGKS-FQIIDTR

WP_055967341.1 DLFHLDLAYAPPFATTKDPVLYTGMALDNAIKKTARLMTPNELIDQVASGKS-FQIIDTR

WP_050677028.1 DLFHLDLAYAPPFATTKDPVLYTGMALDNAIKKTARLMTPNELIDQVASGKS-FQIIDTR

WP_029341349.1 DLFHLDLAYAPPFATTKDPVLYTGMALDNAIKKTARLMTPNELIDQVASGKS-FQIIDTR

WP_026831288.1 DLFHLDLAYAPPFSTTKDPVLYTGMALDNAIKKTARLLTPNELMEQVAGGKS-FQIIDTR

WP_014970324.1 DLFHLDLAYAPPFATTKDPVLYTGMALDNAIKKTARLMTPNELMEQVAGGKS-FQIIDTR

WP_071500702.1 DLFHLDLAYAPPFATTKDPVLYTGMALDNAIKKTARLMTPNELIEQVAGGKS-FQIIDTR

WP_028106360.1 DLFYLDLAYAPPFATTKDPVLYTGMALDNAIKKTARLMTPNELIEQVANGKS-FQIIDTR

WP_026833068.1 DLFHLDLAYAPPFATTKDPVLYTGMALDNAIKKTARLMTPNELIEQVANGKS-FQIIDTR

WP_026828434.1 DLFHLDLAYAPPFATTKDPVLYTGMALDNAIKKTARLMTPNELIEQVANGKS-FQIIDTR

WP_012370312.1 DLFHLDLAYAPPFATTKDPVLYTGMALDNAIKKTARLMTPNELIEQVAGGKS-FQIIDTR

WP_071873852.1 DLFHLDLAYAPPFATTKDPVLYTGMALDNAIKRN-PLMTPTKILAAQEKGEA-IQIIDTR

WP_073414130.1 DLFHLDLAYAPPFSTTKDPVMYTGMALHNSIQNKNRLITPRELIKRIENGEE-LQIIDTR

WP_066448669.1 DLFHLDLAYAPPFSTTKDPVMYTGMALHNAIEKKNKLITPKELSEKIEKGEA-IQVIDTR

WP_016201870.1 DLFHLDLAYAPPFSTTKDPVMYTGMALQNAIEKKNKLITPKELTDRLEKGEK-IQVIDTR

WP_047943166.1 DLFHLDLAYAPPFSTTKDPVMYTGMALQNAIEKKNKLITPKEITERLERGET-IQVIDTR

WP_016204927.1 DLFHLDLAYAPPFSTTKDPVMYTGMALQNAIEKKNKLITPKELTERIEKGEA-LQVIDTR

WP_053602939.1 DLFHLDLAYAPPFSTTKDPVLYTGMALQNAIEKKNKLITPQELTEQIEKGVK-FQIIDTR

WP_010649726.1 DLFHLDLAYAPPFSTTKDPVMYTGMALQNAIDKKNRLITPQELKHRIENEEEKIQVIDTR

WP_074600543.1 DLFHLDLAYAPPFSTTKDPVLYTGMALTNAIEKKNKLMTPQELSDRTESGER-LQIIDTR

WP_008633299.1 DLFHLDLAYAPPFSTTKDPVMYTGMALQNAIDKRNKLMTPQELTERLENGEL-LQVIDTR

WP_027953781.1 DLFHLDLAYAPPFSTTKDPVMYTGMALQNAMDKKNRIITPQELTNRIQNGEK-IQVIDTR

WP_067725917.1 DLFHLDLAYAPPFSTTKDPVMYTGMALQNAIDKKNKLITPEELTDRIESGEQ-LQVIDTR

WP_071649283.1 DLFHLDLAYAPPFSTTKDPVMYTGMALQNAIDKKNKLITPQELTDRIENGEQ-IQIIDTR

WP_010652163.1 DLFHLDLAYAPPFSTTKDPVMYTGMALQNAIDKKNRLITPQELTDKIENGEK-FQIIDTR

WP_041844632.1 DLFHLDLAYAPPFSTTKDPVMYTGMALQNAIDKKNRLITPQELTDRIESGEK-FQIIDTR

WP_050739125.1 ELFNLDLGYAPPFSNTKDPIHYTGMILDNALNNGRDLITSETLKKKLNSEEV-FEIIDTR

SEO96011.1 ELFHLDLAYAPPFSTTKDPVHYTGMILDNAISKERDVRTSSSLD---NPDET-IQIIDTR

WP_069871897.1 ELFHLDLAYAPPFSTTKDPVHYTGMILDNAINRGRKIITSEE----VCSGETAYQIIDAR

OJV63954.1 ELFHLDLAYAPPFSTTKDPIHYTGMILDNAINNGRPIITANEVR---NSESE-LQIVDAR

WP_066068147.1 ELFHLDLAYAPPFSTTKDPVHYTGMILENSISNDRPIITSNEVKSLVSKGEK-IQIVDAR

WP_069999727.1 ELFHLDLAYAPPFSTTKDPVHYTGMILDNALSKGRPIITAKKARELISKGEK-VQVVDAR

SDP09337.1 ELFHLDLAYAPPFSTTKDPVHYTGMILDNALNNNRPIITSKEAQRLIDKGEN-IQVIDAR

WP_023977032.1 ELFHLDLAYAPPFSTTKDPVHYTGMILDNALNNNRPIITSNELKDLVKSSEK-VQIVDAR

SFD29588.1 ELFHLDLAYAPPFSTTKDPVHYTGMILDNALNNNRKIITPKQLRKLVTENEK-VQIVDAR

KAJ53726.1 ELFHLDLAYAPPFSTTKDPVHYTGMILDNALNKNRLTITAKDTENLIKNEEK-IQIIDAR

WP_039633362.1 ELFHLDLAYAPPFSTTKDPVHYTGMILDNAINKGRPIITSKEIERLINKGER-VQIVDAR

WP_017472957.1 DLFHLDLAYAPPYSTTKDPIHYSGMILDNAISKGRPIRTSKDIQAQIDQQQD-IQVIDTR

SFO84805.1 ELFHLDLAYAPPFSTTKDPIHYSGMILDNAMTNNRALKTSEDVRKMQADNQP-LQIIDAR

SFP14577.1 ELFHLDLAYAPPFSTTKDPVHYSGMILDNALSKGRPLKTSDDVRELEANGEK-IQIVDTR

WP_062323720.1 ELFHLDLAYAPPFSTTKDPVHYSGMILDNALSKGRPLKTSDDVRELEANGEK-IQIVDTR

WP_073271217.1 DLFHLDLAYAPPYSTTKDPVMYSGMILDNAISKGRKLLTATQVDALINSGDE-YVIIDAR

WP_031573915.1 DLVHLDLAYAPPFSTTKDPVMYTGMIQENAIYGNRPVMTNKELDELVASGKK-VRIVDAR

WP_074913255.1 DLVHLDLAYAPPFSTTKDPVMYTGMIQENAIYGNRPVMTIKELDELASSGKK-VRIVDAR

OGO93293.1 DLFHLDLAYAPPFSTTKDPVMYSGMILDNAINKNRPLITAKELDTLTKSGEA-YNIIDAR

OJU15484.1 DLFHLDLAYAPPFSTTKDPVMYTGMILDNAISKGRKLMTADELDALMRSGEK-YNLIDVR

SCP98331.1 DLFHLDLAYAPPFSTTKDPVMYTGMILDNAMSQGRKLITADKLAALIESGEG-YQLIDAR

WP_066089511.1 DLFHLDLAYAPPFSTTKDPVLYTGMILDNAIHSDRKLISADDLETLISSSKN-YQLIDAR

SEW43766.1 DLFHLDLAYAPPFSTTKDPVMYTGMILDNAIHKGRPLMTASALKTLMESGEK-YELIDAR

WP_014314980.1 DLFHLDLAYAPPFSTTKDPVMYTGMILDNAIHKGRPLMTAQELDALIDSGEK-YTLIDAR

WP_033166250.1 DLFHLDLAYAPPFSTTKDPVMYTGMILDNAIHKGRPLLTAQGLDELIESGEK-YTLIDAR

WP_066648725.1 DLFHLDLAYAPPFSTTKDPVMYTGMILDNAIHRGRPLITAQELDTLVQSGKP-YTLIDAR

WP_020072493.1 DLFHLDLAYAPPFSTTKDPVMYTGMILDNAIQGGRPLITAKELDALMESGET-YNLIDAR

WP_014314895.1 DLFHLDLAYAPPFSTTKDPVMYTGMILDNAIHRGRPLMTVQELDALMQSGEE-YTLIDAR

WP_023388569.1 DLFHLDLAYAPPFSTTKDPVMYTGMILDNAINGGRSIADPRLIGSGTYDGEE-VQIIDAR

WP_040428848.1 DLFHLDLAYAPPFSTTKDPVMYTGMILTNDIERDRNLISASELSKKINNNEE-ITIIDAR

WP_073016597.1 DLFHLDLAYAPPFSTTKDPVMYTGMVLTNDINTNRKLITPDKLQEKIDNKED-IIIIDTR

WP_021801810.1 DLFHLDLAYAPPFSTAKDPVMYTGMILTNDINSNRKLITPNKLQEKIDNNED-IIIIDTR

WP_018703748.1 DLFHLDLAYAPPFSTTKDPVLYTGMILDNAISRGRKLITSEELARKQQSGAE-VIVIDAR

WP_034869873.1 DLFHLDLAYAPPFSTAKDPVMYTGMILDNAINRDKKLITPREVPKIIEKEKD-VTVIDAR

WP_073538592.1 DLFHLDLAYAPPFSTTKDPVMYTGMILDNVINRDRKLITPNELHKLIESRND-VTVIDAR

WP_007062926.1 DLFHLDLAYAPPFSTTKDPVMYTGMILDNAINRGRKLITPDELNKLIENKSD-VTIIDAR

WP_066620912.1 DLFHLDLAYAPPFSTTKDPVIYTGMILDNAINRGRELITPGELNKLIEKGND-ITVIDAR

WP_004455169.1 DLFHLDLAYAPPFSTTKDPVMYTGMILDNAMNRGRKLITPKELNELRKSGED-VTVIDAR

WP_071167487.1 DLFHLDLAYAPPFSTTKDPVMYTGMILDNAMNRGRKLITPKELNELRKSGED-VTVIDAR

WP_035776858.1 DLFHLDLAYAPPFSTTKDPVMYTGMILDNQINRGRELITPKELNELREAKKD-VIVIDTR

WP_015613982.1 DLFHLDLAYAPPFSTTKDPVMYTGMILDNAINRGRELITPKELNGLRKAGND-VTVIDTR

WP_026883985.1 DLFHLDLAYAPPFSTTKDPVMYTGMILDNAINKRRELITPEELKARIKKGEK-IKIIDAR

WP_007930621.1 DLFHLDLAYAPPFSTTKDPVMYTGMILDNAIRRGRVLMTPAEVQEKIKNGEE-VKIIDAR

WP_007952659.1 DLFHLDLAYAPPFSTTKDPVMYTGMILDNAIRRGRELMTPAELQEKIKNGEE-VKIIDAR

:* ***.*:* ::.:***: *:** * : ::*.*

OHD09048.1 SLEDYE-KGHIPDALHIPFQELKEKSKDFRKSKPIVVHCNKGTTGNAAQNLLINLGFQKV

WP_038668288.1 SNKDFA-KGHIEGAVNIPLGDLKEKVRELSQNESIVVHCNKGVTGNAAQNMLINLGFTKV

WP_026825315.1 KAAQFE-KACVDCAVNVPLGNLREAANDMDKNKPTVVYCNGGVTGNAAQNVLRNLGFTDV

WP_074036424.1 KATQFE-KACVDCAVNVPLGNLREAAKGMDKDKPTVVYCNGGVTGNAAQNVLRNLGFTDV

WP_047794995.1 KASQFE-KACVDCAVNVPLGNLREAAKDMDKDKPTVVYCNGGVTGNAAQNVLRNLGFTDV

WP_074036076.1 KAAQFE-KACVDCAVNVPLGNLREAAKDMDKDKPTVVYCNGGVTGNAAQNVLRNLGFTDV

WP_031422658.1 KASQFE-KACVDCAVNVPLGNLREAAKDMDKDKPTVVYCNGGVTGNAAQNVLRNLGFTDV

WP_012727071.1 KASQFE-KACVDCAVNVPLGNLREAAKDMDKDKPTVVYCNGGVTGNAAQNVLRNLGFTDV

WP_016509108.1 KAVQFE-KACVDCAVNIPLGKLREASKDLDKDKPTVVYCNGGVTGNAAQNVLRNLGFTDV

WP_070328560.1 KAAQFE-KACVDCAVNIPLGKLREASKDLDKDKPTVVYCNGGVTGNAAQNVLRNLGFTDV

WP_071398091.1 KAAQFE-KACVDNAVNIPLGKLREASKDLDKDKPTVVYCNGGVTGNAAQNVLRNLEFTDV

WP_034817875.1 KASQFE-KACVDCAVNIPLGDLRTAAKDMDKDKPTVVYCNGGVTGNAAQNVLRNLGFTDV

WP_034804577.1 KAAQFE-KACVDCAVNVPLGDLRAAAKDMDKNKPTVVYCNGGVTGNAAQNVLRNLGFTDV

WP_047374565.1 KAAQFE-KACVDCAVNVPLGDLRTAAKDMDKDKPTVVYCNGGVTGNAAQNVLRNLGFTDV

WP_029334490.1 KAAQFE-KACVDCAVNVPLGDLRTAAKDMDKDKPTVVYCNGGVTGNAAQNVLRNLGFTDV

WP_024370588.1 KAAQFE-KACVDCAVNVPLGDLRTAAKDMDKDKPTVVYCNGGVTGNAAQNVLRNLGFTDV

WP_021066461.1 KASQFE-KACVDCAVNVPLGDLREAAKDMDKDKPTVVYCNGGVTGNAAQNVLRNLGFTDV

WP_029594929.1 KASQFE-KACVDCAVNIPLGNLRDAAKDMEKDKPTVVYCNGGVTGNAAQNVLRNLGFTDV

WP_058764331.1 KASQFE-KACVDCAVNIPLGDLRAASNDLDKTKPTVVYCNGGVTGNAAQNVLRNLGFTDV

WP_034778796.1 KAAQFE-KACVDCAVNIPLGDLRAAAKDMDKDKPTVVYCNGGVTGNAAQNVLRNLGFTDV

WP_039810825.1 SNAQFE-KAHVKGAVHIPLGDLRTRASELNPDLKTVVYCNKGVTGNAAQNILINHGFKVV

WP_056061304.1 SRAQFE-KSHVDGAIHIPLGELRERAQELDPTVPTIVYCNKGVTGNAAQNVLKNLGFRDV

WP_035408065.1 SRAQFE-KSHVDGAIHIPLGELRERAQELDPTVPTIVYCNKGVTGNAAQNVLKNLGFRDV

WP_029333607.1 SKKQFE-KNHVDGAVHIPLGELRARAAELDPSIPTVVYCNKGVTGNAAQNVLKNLGFTDV

WP_053453049.1 SKAQFE-KNHVDGAIHMPLGELRTRAKELDPTLPTIVYCNKGVTGNAAQNVLKNLGFTDV

WP_058705130.1 SKAQFE-KNHVDGAIHIPLGELRTRAKELDPTLPTIVYCNKGVTGNAAQNVLKNLGFKDV

WP_023467877.1 SKAQFE-KNHVDGAIHIPLGELRTRAKELDPTLPTIVYCNKGVTGNAAQNVLKNLGFTDV

WP_058713610.1 SKAQFE-KNHVDGAIHIPLGELRTRAKELDPTLPTIVYCNKGVTGNAAQNVLKNLGFTDV

WP_058265269.1 SKAQFE-KNHVDGAIHIPLGELRTRAKELDPTLPTIVYCNKGVTGNAAQNVLKNLGFTDV

WP_035397995.1 SKAQFE-KNHVDGAIHIPLGELRTRAKELDPTLPTIVYCNKGVTGNAAQNVLKNLGFTDV

WP_064300131.1 SKAQFE-KNHVDGAIHIPLGELRTRAKELDPTLPTIVYCNKGVTGNAAQNVLKNLGFTDV

WP_055967341.1 SKAQFE-KNHVDGAIHIPLGELRTRAKELDPTLPTIVYCNKGVTGNAAQNVLKNLGFTDV

WP_050677028.1 SKAQFE-KNHVDGAIHIPLGELRIRAKELDPTLPTIVYCNKGVTGNAAQNVLKNLGFTDV

WP_029341349.1 SKAQFE-KNHVDGAIHIPLGELRTRAKELDPTLPTIVYCNKGVTGNAAQNVLKNLGFTDV

WP_026831288.1 SKAQFE-KNHVDGAIHIPLGELRTRAKELDPTLPTIVYCNKGVTGNAAQNVLKNLGFTDV

WP_014970324.1 SKAQFE-KNHVDGAIHIPLGELRTRAKELDPTLPTIVYCNKGVTGNAAQNVLKNLGFTDV

WP_071500702.1 SKAQFE-KNHVDGAIHIPLGELRTRAKELDPTLPTIVYCNKGVTGNAAQNVLKNLGFTDV

WP_028106360.1 SKAQFE-KNHVDGAIHIPLGELRTRAKELDPTLPTIVYCNKGVTGNAAQNVLKNLGFTDV

WP_026833068.1 SKAQFE-KNHVDGAIHIPLGELRTRAKELDSTLPTIVYCNKGVTGNAAQNVLKNLGFTDV

WP_026828434.1 SKAQFE-KNHVDGAIHIPLGELRTRAKELDPTLPTIVYCNKGVTGNAAQNVLKNLGFTDV

WP_012370312.1 SKAQFE-KNHVDGAIHIPLGELRTRAKELDPTLPTIVYCNKGVTGNAAQNVLKNLGFTDV

WP_071873852.1 SKKDYE-KSHVKGAVHIPLGELRIRAEELDKEVPTVTYCNKGVTGNAAQNVLINHGFKEV

WP_073414130.1 VEKQFK-VSHVPGAINIPLSKLRESAKNLHPDLPTITYCNSGVTGNAAQNILINMGIKEV

WP_066448669.1 SPKQYD-FSKVETAINIPLGELREKCTGLNPDLPTVTYCNGGVTGNAAQNVMRNLGFNDI

WP_016201870.1 STEQYK-VSKVESAINIPLGALRDKCKDLNPSLPTVTYCNGGITGNAAQNVLRNLGFYDI

WP_047943166.1 ATKQYN-VSKVESAINIPLGELRKKSKELDPNLPTVTYCNSGVTGNAAQNVLRNLGFNDI

WP_016204927.1 APKQHN-VSKVESAINIPLGELRVKSRELDRNLPTVTYCNGGVTGNAAQNVLRNLGFNDI

WP_053602939.1 STKQYE-VSHVVGAINIPHANLRSEANNLDPEIPTVTYCNKGVTGNAAQNVLRNMGFKNV

WP_010649726.1 ASKQFE-ASNVKGAINIPLANLRTKAKDLDPDIPTVTYCNSGVSGNAAQNVLRNMGFKEV

WP_074600543.1 AAKQFE-VSNVEGAVNIPLANLREEAEGLDPDVPTVTYCNKGVTGNAAQNVLRNKGFKEV

WP_008633299.1 ATKQFE-VSNVPGAVNIPLAELRSKAKNLDPDIPTVTYCNSGVTGNAAQNVLRNMGFKEV

WP_027953781.1 APKQYE-VSNVEGAVNIPLAELRAEAKQLDPAIPTVTYCNKGVTGNAAQNVLINMGFEEV

WP_067725917.1 ASKQFE-VSNVAGAINIPLANLRTEAKNLNPDIPTVTYCNSGVTGNAAQNVLRNMGFKEV

WP_071649283.1 ATKQYE-VSKVDGAVNMPLAKLRKEAKNLDPNIPTVTYCNKGVTGNAAQNVLINMGFKDV

WP_010652163.1 ATKQYE-VSNVDGAINIPLAELRSEGKKLNPNLPTVTYCNKGVTGNAAQNVLTNMGFKEV

WP_041844632.1 ATKQYE-VSNVDGAINIPLAKLRSEAQNLDPNLPTVTYCNSGVTGNAAQNVLRNLGFKEV

WP_050739125.1 IEQQYQ-DSHIPNAKNIPHSELRGRLSMLNKSAPIVTYCNKGVTGNATQNILINHGFKKV

SEO96011.1 TKQQYN-EGHVKESINIPHAEIRERMGELDKEVPVLTYCNGGTTGNAVQNILINHGFKNV

WP_069871897.1 SLEDFETKGHVKGAIHIPHAELRVALKKLDPEKPTVTYCNKGVTGNAAQNILINKGFKSV

OJV63954.1 SSEDYDIKGHVTGAVNMPHAKLRDVLKNLDPEKPTVTYCNKGVTGNAAQNILINNGFKKV

WP_066068147.1 VSKQYE-ESHVDNAINIPHANLRDNLDSLDKDTVTVTYCNKGVTGNAAQNILINHGFKKV

WP_069999727.1 VSKQYE-EARVDTAVNMPHSKLREEIKKLDKEIPTITYCNKGVTGNAAQNILINEGFKEV

SDP09337.1 ISKQYD-ENHVYTAINIPHSKLRNELKTLDKNVLTVTYCNKGVTGNATQNILINHGFKNV

WP_023977032.1 VSKQYD-DAHVDNAVNIPHKNLRNEIENLDKDAVVVTYCNKGVTGNAAQNIFINHGYKKV

SFD29588.1 VSKQYD-QAHVEDAVNIPHSNLRESMENVDKDSVVVTYCNKGVTGNATQNIFINHGYKKV

KAJ53726.1 DSKQYT-ESHVDSAINIPHSHLREKLKELDKNITTITYCNKGVTGNAAQNILINHGFKKV

WP_039633362.1 VNKQYD-ESHVDTAINIPHSNLRNELDTLDKDILTITYCNKGVTGNAAQNILINYGFKKV

WP_017472957.1 VTKQYD-TAHVDSAVNIPHADIRAKLAELDKHKPTITYCNSGTTGNAVQNILLNHGFTDV

SFO84805.1 AEKQHI-VNCVDGAISMPHAIVRARLNELDKNLPTVTYCNSGTTGNAVQNILINYGFKDV

SFP14577.1 IEKQHL-ANCVDGAVSMPHAVIRARLNELDKNLPTVTYCNSGTTGNAVQNILLNHGFNDV

WP_062323720.1 IEKQHL-ANCVDGAVSMPHAVIRARLNELDKNLPTVTYCNSGTTGNAVQNILLNHGFKDV

WP_073271217.1 VEGQYQ-KHHIPTAVNIPHNELRERLGEVDKEKIAVTYCNKGVTGNAAQNILICNGFEKV

WP_031573915.1 VIKQYE-ASHVENAINVPHSAARDFAKDLEKDTITVTYCNKGTTGNAVQNIFLNSGVEHV

WP_074913255.1 VVKQYE-ASHVENAINVPHSAARDVAKDLEKDTITVTYCNKGTTGNAVQNIFLNSGVEKV

OGO93293.1 VATQYE-KSHIDTAHNLPHQEIRSCCDSLDKDTLTVTYCNKGVTGNATQNILLNKGFKKV

OJU15484.1 ASEQFN-KKHMDTAQNIPHAALRNAVDTMDKNTVTVTYCNKGVTGNAAQNILLNNGFRKV

SCP98331.1 VSAQYE-KDHIETAVNLPHGELREQLKDLNPEAVTITYCNKGVTGNAAQNILLNHGFQEV

WP_066089511.1 EASQYE-ENHVETAMSIPHAKLRDALDDLNKETMTITYCNKGVTGNAAQNILLNHGFKEV

SEW43766.1 AAGQYE-KAHVENAENIPHAKLRVSLKSLDKEVVAITYCNKGVTGNAAQNILINAGFKKV

WP_014314980.1 AVAQYD-KNHIETAKSIPHAKLRAVVESLDKDAVAVTYCNKGVTGNAAQNILLGRGLKKV

WP_033166250.1 VVTQYE-KNHIESAKSIPHSKLRSELPDIEQEEVVVTYCNKGVTGNAAQNILMGRGLKKV

WP_066648725.1 AAAQYE-QGHIETAKSIPHAKVRATAESLDRDVITVTYCNKGVTGNAAQNILINDGFRKV

WP_020072493.1 VAGQYD-KGHIDNAQSIPHSKLRDAADELDPDAIAVTYCNKGVTGNAAQNILIGKGYKKV

WP_014314895.1 VSAQYE-KNHVDTAESIPHEKLRASSETMDKDAITVTYCNKGTTGNAAQNILLGKGFKKV

WP_023388569.1 AESQYQ-EGHVEGAVSMPHETVRGKLEGLDREKPVIAYCNKGTTGNAVQNILLNSGFKMV

WP_040428848.1 VAKQYN-ESHVEGAINVPHERLRDTLESLDKDKSIVTYCNKGVTGNAAQNILINSGFKKV

WP_073016597.1 EPKQYE-VSHVDEAINIPHKDLREKANSLDKDKLTVTYCNKGVTGNATQNILLNMGFKDV

WP_021801810.1 EPKQYE-VSHVDGAINIPHKELRDKANTLDKEKLTITYCNKGVTGNATQNILLNMAFKNV

WP_018703748.1 AAGQYE-KDHVQEALNIPQEKLREAAEALDKTKMIVTYCNKGVTGNAAQNILLNRGFAQV

WP_034869873.1 VEKQYE-EAHIEDAINIPHECIRESCNKLDKEKTIITYCNKGVTGNAVQNILINNGFKKV

WP_073538592.1 VCRQFE-ESHVDNAINIPHDEVRGKVNQLDKEKPIVAYCNKGVTGNAVQNILMNNGCKKV

WP_007062926.1 VNKQYE-EVHVNNAINIPHDQIREKADELNKEKIIVTYCNKGVTGNAAQNILINKGFKKV

WP_066620912.1 VNKQYE-EAHVDTAINIPHGQIRKKAEELDKEKVVVTYCNKGVTGNAAQNILINQGFKKV

WP_004455169.1 VKRQYE-EAHVENAISIPHGEFRDNVKNLNKDKICVTYCNKGVTGNAAQNILINSGFKKV

WP_071167487.1 VKRQYE-EAHVENAISIPHGEFRDNVKNLNKDKICVTYCNKGVTGNAAQNILINSGFKKV

WP_035776858.1 VSKQYA-EAHVENAINIPHSEFRDKTKELDKDKIFVTYCNKGVTGNATQNILINNGFKKV

WP_015613982.1 VSRQYE-EAHVENAINIPHAQVRNKIDELDKEKVVVTYCNKGVTGNAVQNILINNGFKKV

WP_026883985.1 VSGQYE-AGHVEESENIPQDKIRGILDGLDKDEITVTYCNKGVTGNAVQNILINNKFKKV

WP_007930621.1 VTKQYE-KAHVEGAVNIPQENIRKALDSLDKEMVTVTYCNKGVTGNAAQNILINHGFKNV

WP_007952659.1 VTKQYE-KAHVEGAVNIPQENIRKALDSLDKEMVTVTYCNKGVTGNAAQNILINHGFKNV

:. : : :* : . :.:** * :***.**:: :

OHD09048.1 YNLSGGYQNWKNQENL---------

WP_038668288.1 YNLSGGYKEFKVENQ----------

WP_026825315.1 YNLSGGNKNYQLFKKYGN-------

WP_074036424.1 YNLSGGNKNYQLFKKYGN-------

WP_047794995.1 YNLSGGNKNYQLFKKYGN-------

WP_074036076.1 YNLSGGNKNYQLFKKYGN-------

WP_031422658.1 YNLSGGNKNYQLFKKYGN-------

WP_012727071.1 YNLSGGNKNYQLFKKYGN-------

WP_016509108.1 YNLSGGNKNYQLFKKYGN-------

WP_070328560.1 YNLSGGNKNYQLFKKYGN-------

WP_071398091.1 YNLSGGNKNYQLFKKYGN-------

WP_034817875.1 YNLSGGNKNYQLFKKYGN-------

WP_034804577.1 YNLSGGNKNYQLFKKYGN-------

WP_047374565.1 YNLSGGNKNYQLFKKYGN-------

WP_029334490.1 YNLSGGNKNYQLFKKYGN-------

WP_024370588.1 YNLSGGNKNYQLFKKYGN-------

WP_021066461.1 YNLSGGNKNYQLFKKYGN-------

WP_029594929.1 YNLSGGNKNYQLFKKYGN-------

WP_058764331.1 YNLSGGNKNYQLFKKYGN-------

WP_034778796.1 YNLSGGNKNYQLFKKYGN-------

WP_039810825.1 YNLSGGNKNYQHVVNNE--------

WP_056061304.1 CNLSGGNQNYQHIKSCL--------

WP_035408065.1 CNLSGGNQNYQHIKSCL--------

WP_029333607.1 CNLSGGNTNYQHLKHCFPKK-----

WP_053453049.1 CNLSGGNKNYQHIKKCLS-------

WP_058705130.1 CNLSGGNKNYQHIKKCLS-------

WP_023467877.1 CNLSGGNKNYQHIKKCLS-------

WP_058713610.1 CNLSGGNKNYQHIKKCLS-------

WP_058265269.1 CNLSGGNKNYQHIKKCLS-------

WP_035397995.1 CNLSGGNKNYQHIKKCLS-------

WP_064300131.1 CNLSGGNKNYQHIKKCLS-------

WP_055967341.1 CNLSGGNKNYQHIKKCFS-------

WP_050677028.1 CNLSGGNKNYQHIKKCFS-------

WP_029341349.1 CNLSGGNKNYQHIKKCFS-------

WP_026831288.1 CNLSGGNKNYQHIKKCLAN------

WP_014970324.1 CNLSGGNKNYQHIKKCLAN------

WP_071500702.1 CNLSGGNKNYQHCKKFLAN------

WP_028106360.1 CNLSGGNKNYQHCKKCLAD------

WP_026833068.1 CNLSGGNKNYQHCKKCLAD------

WP_026828434.1 CNLSGGNKNYQHCKKCLTE------

WP_012370312.1 CNLSGGNKNYQHCKKCLTE------

WP_071873852.1 YNLSGGNENYQSYVAMLDAIK----

WP_073414130.1 YNLSGGNKNFQSNLKN---------

WP_066448669.1 YNLSGGNKNYQNYMKNK--------

WP_016201870.1 YNLSGGNKNYQNYMKNK--------

WP_047943166.1 YNLSGGNKNYQNYMKNK--------

WP_016204927.1 YNLSGGNKNYQNYMKNK--------

WP_053602939.1 YNLSGGNKNYQKFINN---------

WP_010649726.1 YNLSGGNKNYQSFSKN---------

WP_074600543.1 YNLSGGNKNYQRFNKDK--------

WP_008633299.1 FNLSGGNKNYQIFRSF---------

WP_027953781.1 YNLSGGNKNYQSFSKANQ-------

WP_067725917.1 FNLSGGNKNYQSFHKQ---------

WP_071649283.1 FNLSGGNKNYQSFNKGKQ-------

WP_010652163.1 FNLSGGNKNYQSFKKGNK-------

WP_041844632.1 FNLSGGNKNYQSFNNGK--------

WP_050739125.1 YNLSGGYEFFEATKEK---------

SEO96011.1 SNLSGGYKFYRTTLAE---------

WP_069871897.1 FNLSGGHKFYKGSGHNE--------

OJV63954.1 FNLSGGHKFYKGSGEQK--------

WP_066068147.1 YNLSGGHKFYKATKK----------

WP_069999727.1 YNLSGGHKFYKATKGE---------

SDP09337.1 CNLSGGHKFYKSTKK----------

WP_023977032.1 YNLSGGHKFYKGSKK----------

SFD29588.1 YNLSGGYKFYKGSK-----------

KAJ53726.1 YNLSGGHKFYKSIKKI---------

WP_039633362.1 YNLSGGHKFYNAIKNKQ--------

WP_017472957.1 SNLSGGHKFYRATKKDR--------

SFO84805.1 SNLSGGNKFYQATKKKQDK------

SFP14577.1 SNLSGGNKFYQATKKTRDQ------

WP_062323720.1 SNLSGGNKFYQATKKTRDQ------

WP_073271217.1 YSVSGGHKTYKRYMEKKKK------

WP_031573915.1 YNLSGGHKTYLRYLKMKKK------

WP_074913255.1 YNLSGGHKTYLRYLKMKKK------

OGO93293.1 YNLSGGFKQYKITHKKSDF------

OJU15484.1 FNVSGGQKQYNAQNKE---------

SCP98331.1 YNISGGHKQFRKVRK----------

WP_066089511.1 YNLSGGHKNYSKNKKSF--------

SEW43766.1 YNLSGGHKQYSKSMKI---------

WP_014314980.1 YNLSGGQKQYSKTHNKK--------

WP_033166250.1 YNLSGGQKQYSKTHSKSKK------

WP_066648725.1 YNLSGGHKHYCKMHSEK--------

WP_020072493.1 FNLSGGHKQYKNTHKE---------

WP_014314895.1 YNLSGGQKQYGILHEE---------

WP_023388569.1 YNISGGNTNYRTQKKKRD-------

WP_040428848.1 YNLSGGYKNYSKTVKNQS-------

WP_073016597.1 YNLSGGNKNYSKTVKK---------

WP_021801810.1 YNLSGGNKNYSKILKK---------

WP_018703748.1 YNLSGGHKQYQRIQQHLSRQEDPGK

WP_034869873.1 YNISGGYNNYKIQKKE---------

WP_073538592.1 YNISGGHENYKTQTN----------

WP_007062926.1 YNISGGHKNYKAQTK----------

WP_066620912.1 YNISGGHKNYKAQSK----------

WP_004455169.1 YNLSGGHKNYKAFENK---------

WP_071167487.1 YNLSGGHKNYKAFENK---------

WP_035776858.1 YNLSGGHKNYKAFENKLIK------

WP_015613982.1 YNISGGHKNYKAQTK----------

WP_026883985.1 YNLSGGFKNYKNGSPK---------

WP_007930621.1 YNLSGGYKNYSKNSKK---------

WP_007952659.1 YNLSGGYKNYSKNSKK---------

.:*** :
